# Proteolytically generated soluble Tweak Receptor Fn14 is a blood biomarker for γ‐secretase activity

**DOI:** 10.15252/emmm.202216084

**Published:** 2022-09-07

**Authors:** Gökhan Güner, Marlene Aßfalg, Kai Zhao, Tobias Dreyer, Shibojyoti Lahiri, Yun Lo, Bianca Ionela Slivinschi, Axel Imhof, Georg Jocher, Laura Strohm, Christian Behrends, Dieter Langosch, Holger Bronger, Christopher Nimsky, Jörg W Bartsch, Stanley R Riddell, Harald Steiner, Stefan F Lichtenthaler

**Affiliations:** ^1^ German Center for Neurodegenerative Diseases (DZNE) Munich Germany; ^2^ Neuroproteomics, School of Medicine, Klinikum rechts der Isar Technical University of Munich Munich Germany; ^3^ Department of Neurosurgery Philipps University Marburg Marburg Germany; ^4^ Department of Gynecology and Obstetrics, School of Medicine, Klinikum rechts der Isar Technical University of Munich Munich Germany; ^5^ Protein Analysis Unit, Faculty of Medicine Biomedical Center, LMU Martinsried Germany; ^6^ Immunotherapy Integrated Research Center Fred Hutchinson Cancer Research Center Seattle WA USA; ^7^ Munich Cluster for Systems Neurology (SyNergy), Medical Faculty LMU Munich Germany; ^8^ Technical University of Munich Freising Germany; ^9^ German Cancer Consortium (DKTK), partner site Munich and German Cancer Research Center (DKFZ) Heidelberg Germany; ^10^ Department of Medicine University of Washington Seattle WA USA; ^11^ Division of Metabolic Biochemistry, Faculty of Medicine, Biomedical Center (BMC) LMU Munich Germany; ^12^ Munich Cluster for Systems Neurology (SyNergy) Munich Germany

**Keywords:** Alzheimer's disease, ectodomain shedding, glioblastoma, intramembrane proteolysis, TNR12, Biomarkers, Cancer

## Abstract

Fn14 is a cell surface receptor with key functions in tissue homeostasis and injury but is also linked to chronic diseases. Despite its physiological and medical importance, the regulation of Fn14 signaling and turnover is only partly understood. Here, we demonstrate that Fn14 is cleaved within its transmembrane domain by the protease γ‐secretase, resulting in secretion of the soluble Fn14 ectodomain (sFn14). Inhibition of γ‐secretase in tumor cells reduced sFn14 secretion, increased full‐length Fn14 at the cell surface, and enhanced TWEAK ligand‐stimulated Fn14 signaling through the NFκB pathway, which led to enhanced release of the cytokine tumor necrosis factor. γ‐Secretase‐dependent sFn14 release was also detected *ex vivo* in primary tumor cells from glioblastoma patients, in mouse and human plasma and was strongly reduced in blood from human cancer patients dosed with a γ‐secretase inhibitor prior to chimeric antigen receptor (CAR)‐T‐cell treatment. Taken together, our study demonstrates a novel function for γ‐secretase in attenuating TWEAK/Fn14 signaling and suggests the use of sFn14 as an easily measurable pharmacodynamic biomarker to monitor γ‐secretase activity *in vivo*.

## Graphical abstract

The Tweak receptor Fn14 has a central role in tissue homeostasis, injury and tumors. The new study demonstrates that the protease γ‐secretase cleaves Fn14 within its transmembrane domain and controls Fn14 abundance, turnover and signaling. This study also establishes soluble, cleaved Fn14 as a biomarker for γ‐secretase activity *in vivo*.

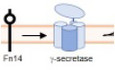

**The paper explained**

1 **Problem**

The protease γ‐secretase is a major drug target for Alzheimer's disease and Notch‐dependent tumors, but therapeutic γ‐secretase inhibition is associated with mechanism‐based side effects. A major challenge for monitoring and adjusting γ‐secretase inhibition is the lack of a suitable, easily measurable *in vivo* pharmacodynamic marker for γ‐secretase activity.

**Results**

We identify the cell surface TWEAK receptor Fn14 as a novel substrate that is directly cleaved by γ‐secretase within its transmembrane domain. The study establishes γ‐secretase cleavage as a novel mechanism controlling abundance and signaling of Fn14. Inhibition of γ‐secretase reduced release of the soluble Fn14 cleavage product into the conditioned medium of cultured cells and primary tumor cells *in vitro* and into blood of mice and humans.

**Impact**

The soluble Fn14 cleavage product is useful as an easily detectable pharmacodynamic activity marker for γ‐secretase that may be instrumental for developing a new generation of γ‐secretase‐targeted drugs with enhanced specificity and a better safety profile. Given the major role of Fn14 in tissue homeostasis, injury, and chronic diseases, the newly discovered proteolytic cleavage of Fn14 by γ‐secretase may also offer new therapeutic options for modulating Fn14 function in disease.

## Introduction

**Figure 1:**
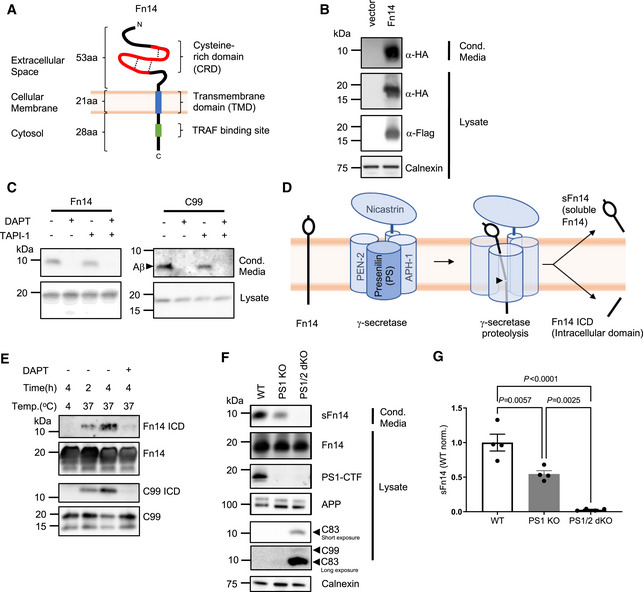
Fn14 is processed by the protease γ‐secretase Domain structure of Fn14. Fn14 has a short, compact N‐terminal extracellular domain. It consists of 53 amino acids that contain three disulfide bridges (dashed lines) that form the cysteine‐rich domain, CRD (red). Fn14 has a single transmembrane domain (blue) and a short C‐terminal cytosolic tail with a TRAF binding site (green) important for its signaling function. Protein N‐ and C‐termini (N, C) are indicated.Detection of sFn14 in conditioned media. HEK293E cells were transfected with either empty vector or a plasmid encoding human Fn14 that bears an N‐terminal HA‐tag and a C‐terminal double FLAG‐tag. Conditioned media and lysates of the transfected cells were collected and analyzed by immunoblotting with the indicated antibodies. Calnexin served as a loading control. Shown are representative blots from *N* = 3 experiments.Generation of sFn14 is sensitive to the γ‐secretase inhibitor DAPT. HEK293E cells were transfected with either Fn14 or C99 (C‐terminal fragment of APP), both containing a N‐terminal HA‐tag and C‐terminal double FLAG‐tag. One day after transfection, cells were treated with γ‐secretase inhibitor DAPT (1 μM), broad‐spectrum metalloprotease inhibitor TAPI‐1 (50 μM), or the corresponding amount of vehicle DMSO as indicated. The conditioned media and the lysates were blotted with anti‐HA antibody. Shown are representative blots from *N* = 4 experiments.Schematic representation of Fn14 shedding by the γ‐secretase complex. γ‐Secretase is a hetero‐tetrameric complex consisting of the indicated subunits, with presenilin being the catalytic subunit and the ectodomain of nicastrin forming a lid‐like structure on top of the γ‐secretase complex. Proteolysis takes place at the catalytic core (indicated by black arrow head), within the lipid bilayer. Proteolysis results in one fragment released into the extracellular space (sFn14), and one fragment into the cytosol (Fn14 ICD, intracellular domain).
*In vitro* γ‐secretase cleavage assay. HEK293E cells were transfected with epitope‐tagged Fn14 or C99. Cellular membranes were collected and incubated under indicated conditions for the γ‐secretase activity assay. Reactions were terminated and ultracentrifuged. Supernatant (containing γ‐secretase cleavage products) was used for detecting the ICD fragment while the pellet was used to blot for full‐length proteins Fn14 or C99. Blotting is done by anti‐FLAG antibody. Shown are representative blots from *N* = 3 experiments.sFn14 production requires the proteolytic presenilin subunit of γ‐secretase. HEK293 cells stably transfected with APP carrying the Swedish double mutation (K595N/M596L) and with a CRISPR/CAS9‐mediated knockout of presenilin 1 (PS1 KO) or of both PS1 and PS2 (PS1/2 dKO), were transiently transfected with epitope‐tagged Fn14. Conditioned media and lysates of the transfected cells were blotted with the indicated antibodies. C83 is a small C‐terminal fragment of APP (containing the C‐terminal 83 amino acids), which is also subjected to γ‐secretase processing. Upon longer exposure, the C99 (generated by BACE1 from APP) is also visible in the PS1/2 dKO condition. Shown are representative blots from *N* = 4 experiments.Quantification of results from panel (F). sFn14 levels in the conditioned media quantified and normalized to WT sFn14 signal.Data Information: Panel (G) shows mean ± SEM, along with *P*‐values calculated by ordinary one‐way ANOVA test with Tukey's multiple comparison test. Number of biological replicates performed is indicated in the corresponding panel legend.

Cell surface receptors have fundamental functions in signaling and communication within a tissue. Since dysregulation of cell surface receptor signaling can lead to disease, the abundance and activity of the receptors are tightly regulated, including by posttranslational mechanisms, such as limited proteolysis (Lichtenthaler *et al*, 2018). However, the regulatory mechanisms are often only partly understood, including for Fn14 (also known as TWEAK receptor, TNR12, TNFRSF12a, and CD266), a cell surface receptor with key functions in tissue homeostasis and tissue injury (Winkles, 2008; Wajant, 2013; Poveda *et al*, 2021). Fn14 is a type I transmembrane protein and the smallest member of the tumor necrosis factor (TNF) receptor superfamily (Meighan‐Mantha *et al*, 1999; Aggarwal, 2003), with an extracellular domain (ectodomain) of only 53 amino acids (Fig 1A). Binding of the cytokine TWEAK (tumor necrosis factor‐like weak inducer of apoptosis) to Fn14 leads to the activation of intracellular signaling pathways, in particular the canonical and noncanonical NFκB pathways (Burkly *et al*, 2011; Poveda *et al*, 2021).

While TWEAK is ubiquitously expressed (Chicheportiche *et al*, 1997), Fn14 protein abundance is low in healthy tissues. Upon tissue injury or cell stress, Fn14 abundance increases rapidly (Meighan‐Mantha *et al*, 1999; Feng *et al*, 2000; Vendrell *et al*, 2010), in particular on the surface of epithelial, endothelial, and other nonhematopoietic cells. Fn14 upregulation helps to mediate local tissue responses including tissue remodeling and inflammation, for example during burn wound repair (Liu *et al*, 2019), but also controls proliferation, apoptosis, cell migration, and differentiation (Winkles, 2008; Poveda *et al*, 2021) and synapse function in the visual system (Cheadle *et al*, 2020). However, increased Fn14 levels or signaling are also linked to chronic diseases, such as most solid tumor types, acute kidney disease, fibrosis, Alzheimer's disease, and cerebral ischemia (Cheng *et al*, 2013; Perez *et al*, 2016; Hu *et al*, 2017; Wang *et al*, 2017; Connolly *et al*, 2021; Nagy *et al*, 2021). Thus, Fn14 is intensively tested as a drug target using monoclonal antibodies or immunotoxins targeted to Fn14 or fusion proteins encoding Fn14 decoy receptors, which improve pathology in various mouse models of disease, such as for lung tumors, triple‐negative breast cancer, tumor cachexia, and atherosclerosis (Yepes *et al*, 2005; Schapira *et al*, 2009; Michaelson *et al*, 2011; Wajant, 2013; Zhou *et al*, 2014; Johnston *et al*, 2015; Peng *et al*, 2018; Alvarez de Cienfuegos *et al*, 2020; Dancy *et al*, 2020; Wolf *et al*, 2021).

Despite the major roles of Fn14 in healthy, injured, and diseased tissues, relatively little is known about the mechanisms that control abundance and, thus, function of Fn14 at the cell surface. Fn14 is constantly synthesized, but rapidly degraded in healthy conditions and has a short half‐life of only about 70 min in cultured HeLa cells (Gurunathan *et al*, 2014). Lysosomal pathways and autophagy contribute to Fn14 degradation (Gurunathan *et al*, 2014; Winer *et al*, 2018). Additional mechanisms are possible, but have not been explored in detail.

One cellular process controlling the abundance and function of numerous membrane proteins, including cell surface receptors, is proteolytic ectodomain shedding, where a protease cleaves a membrane protein within the extracellular or luminal juxtamembrane domain or even within the transmembrane domain (Lichtenthaler *et al*, 2018). As a result, the protein's soluble ectodomain is secreted into the conditioned medium of cultured cells *in vitro* or into body fluids *in vivo*. A soluble form of Fn14 (sFn14) was detected in serum and urine of a mouse model of the kidney disease nephrotoxic nephritis and in serum of healthy humans, but it remained unclear whether this sFn14 comprises only the ectodomain and, thus, results from proteolytic ectodomain shedding or represents full‐length Fn14, potentially released from multivesicular bodies or dead cells (Sharif *et al*, 2016; Chen *et al*, 2018, 2019).

Here, we investigated whether Fn14 undergoes ectodomain shedding and whether such proteolytic cleavage constitutes a mechanism controlling abundance and signaling of Fn14. We report that Fn14 undergoes proteolytic ectodomain shedding *in vitro* and *in vivo*, releasing sFn14 into plasma of mice and humans. We find that this cleavage occurs within the transmembrane domain of Fn14 and is mediated by the intramembrane protease γ‐secretase, which has key roles in development through cleavage of the Notch receptor and in Alzheimer's disease, where it contributes to generation of the pathogenic amyloid β (Aβ) peptide (De Strooper *et al*, 1998, 1999; Wolfe *et al*, 1999; Güner & Lichtenthaler, 2020). We report that γ‐secretase cleavage of Fn14 constitutes a new mechanism of attenuating TWEAK/Fn14 signaling. Finally, we demonstrate the utility of sFn14 as a pharmacodynamic biomarker to monitor γ‐secretase activity *ex vivo* and *in vivo*, including in human tumor patients.

## Results

### Fn14 is shed by the protease γ‐secretase

To determine whether Fn14 is proteolytically processed, we used human embryonic kidney 293 (HEK293) cells transiently transfected with a human Fn14 expression construct carrying an HA‐epitope tag at the extracellular N‐terminus and a double FLAG‐epitope tag at the intracellular C‐terminus (for scheme of Fn14 domain structure see Fig 1A). Using immunoblots, sFn14 was detected with the anti‐HA antibody in the conditioned medium of the transfected cells, but not in the medium of cells transfected with the empty vector (Fig 1B). The apparent molecular weight of the tagged sFn14 was approx. 10 kDa, which is about half of the mass of full‐length, epitope‐tagged Fn14, which was detected by both the anti‐HA and the anti‐FLAG antibodies with a molecular weight of 19 kDa in the lysate (Fig 1B), indicating that sFn14 may be the proteolytically shed cleavage product of full‐length Fn14. We conclude that sFn14 is an N‐terminal proteolytic cleavage product, likely to result from Fn14 shedding.

To identify the protease—referred to as sheddase—which sheds Fn14, we first used a pharmacological approach and tested TAPI‐1, a broad‐spectrum metalloprotease inhibitor that blocks several members of the matrix metalloprotease (MMP) and the “a disintegrin and metalloprotease” (ADAM) families, including ADAM10 and ADAM17, which mediate the shedding of numerous membrane proteins (Dreymueller *et al*, 2015; Saftig & Lichtenthaler, 2015; Zunke & Rose‐John, 2017; Hsia *et al*, 2019). Because Fn14 has a type I membrane orientation (N‐terminus luminal/outside), we also tested DAPT, an inhibitor of γ‐secretase (Dovey *et al*, 2001), which commonly requires its substrates to have a transmembrane domain with type I membrane orientation. Although ADAM proteases shed several TNFRs (Peschon *et al*, 1998; Reddy *et al*, 2000; Weskamp *et al*, 2004; Colombo *et al*, 2018), TAPI‐1 did not block sFn14 generation (Fig 1C). By contrast, the γ‐secretase inhibitor DAPT completely blocked secretion of sFn14 from transfected HEK293 cells (Fig 1C). As a control, DAPT also blocked secretion of the Alzheimer's disease‐linked Aβ peptide from HEK293 cells transfected with C99 (Fig 1C), a C‐terminal fragment of the amyloid precursor protein (APP) that is an established substrate for γ‐secretase (Lichtenthaler *et al*, 1999).

As a result of γ‐secretase cleavage, the intracellular domain (ICD) of Fn14 should also be detectable (Fig 1D). Because ICDs are generally short‐lived and difficult to detect in the cell lysate (e.g., Edbauer *et al*, 2002), we used an established *in vitro* assay (Sastre *et al*, 2001), in which membranes prepared from transfected HEK293 cells were incubated at 37°C to allow for *de novo* production of the Fn14 ICD. This treatment led to a time‐dependent increase in the generation of the Fn14 ICD (Fig 1E), which was γ‐secretase‐dependent, as it was blocked with DAPT. Similar results were obtained for ICD generation from the APP‐derived C99 (Fig 1E).

To validate the pharmacological results with a genetic approach, we transfected Fn14 into APP‐expressing HEK293 cells with a knockout of γ‐secretase activity (Tagami *et al*, 2017). γ‐Secretase is a ubiquitously expressed, membrane‐embedded, hetero‐tetrameric complex that resides within the membranes of the secretory and endocytic pathways (Fig 1D for scheme) (Edbauer *et al*, 2003; Meckler & Checler, 2016; Sannerud *et al*, 2016). The proteolytic subunit of γ‐secretase is presenilin (PS) that contains the active‐site aspartyl residues and exists as two homologs, PS1 and PS2 (Steiner *et al*, 1999; Wolfe *et al*, 1999). Compared with control‐transfected HEK293 cells, sFn14 generation was reduced to about half (54.5 ± 4.9%) after a CRISPR/Cas9‐mediated knockout of PS1 and nearly completely prevented (2.8% ± 0.7 of control cells) in HEK293 cells lacking both PS1 and PS2 (Fig 1F and G). As a control, we blotted for C83, the C‐terminal fragment of APP that lacks the APP ectodomain as a result of APP‐cleavage by ADAM10 (Kuhn *et al*, 2010). C83 is similar to C99, but lacks the N‐terminal 16 amino acids of C99. After its generation, C83 is rapidly cleaved by γ‐secretase so that it accumulated in the PS1 and PS2 double knockout cells (Fig 1F).

Taken all results together, we conclude that Fn14 undergoes shedding by γ‐secretase.

### The cleavage site by γ‐secretase is located within the transmembrane domain of Fn14

**Figure 2:**
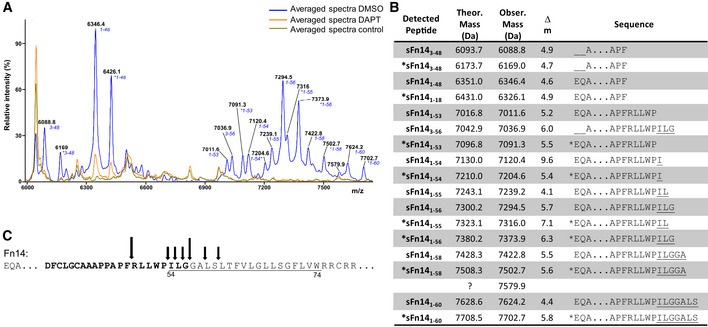
γ‐Secretase cleavage site of Fn14 is located within its transmembrane domain Using an anti‐HA‐epitope antibody, sFn14 was immunoprecipitated from the conditioned medium of HEK293E cells that were untransfected (Control) or transiently transfected with an HA‐tagged Fn14‐expressing construct and additionally treated overnight with DMSO or DAPT (1 μM). The *m*/*z* values of each peak are indicated as well as—above them—the amino acid numbers of the peptide fragment corresponding to the amino acid numbers of wild‐type Fn14. The asterisk (*) labels the peaks that show a mass shift that can be explained by phosphorylation of the annotated fragment. Data are averaged from *N* = 4 experiments.Detected sFn14 peptides generated by γ‐secretase are listed with their theoretical (theor.) and observed (obser.) mass and the mass difference (Δ*m*) between theoretical and observed mass. The corresponding peptide sequence is also indicated. “*” indicates the peptides with the mass shift that corresponds to addition of a phosphate group to the peptide. The underlined text highlights the transmembrane domain of Fn14 as annotated in UniProt. Three intact disulfide bridges are included in the mass calculations, while methionines were considered nonoxidized in the theoretical mass.Schematic representation of the γ‐secretase cleavage sites in Fn14. The underlined sequence indicates the transmembrane domain of Fn14, according to Uniprot. Arrows indicate the cleavage sites. The larger arrows show the cleavage sites with the higher intensity after amino acids 48 and 56. Numbers of amino acids refer to full‐length Fn14 after signal peptide cleavage, which removes the first 27 amino acids of the Uniprot‐annotated sequence. The C‐terminal peptide (DF…ILG) which was generated upon AspN cleavage and used for fragmentation is highlighted in bold.

**Figure EV1:**
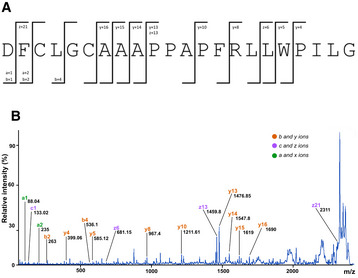
Fragmentation spectrum and sequencing of C‐terminal sFn14 peptide sFn14, immunoprecipitated from the conditioned medium of HA‐Fn14‐trasfected HEK293E cells, was digested with AspN. The peptide corresponding to the C‐terminal fragment with the sequence “DFCLGCAAAPPAPFRLLWPILG” was fragmented and sequenced. Detected fragment ions are schematically indicated.Fragmentation spectrum of the peptide “DFCLGCAAAPPAPFRLLWPILG.” Ion names and measured masses are indicated in the figure and are representative for *N* = 2 experiments.

γ‐Secretase cleaves its substrates within their transmembrane domains (Güner & Lichtenthaler, 2020). To determine whether this is also true for Fn14, we immunoprecipitated sFn14 from the conditioned medium of HEK293 cells transfected with N‐terminally HA‐epitope‐tagged Fn14 and analyzed it by MALDI‐TOF mass spectrometry. We detected several peaks in the range of 6,000–7,700 *m*/*z* corresponding to sFn14 molecules with mostly the same N‐terminus generated through signal peptide cleavage but having different C‐termini, ending within the Fn14 TMD (mass range > 7,000 *m*/*z*) or just before (mass range < 7,000 *m*/*z*) (Fig 2A–C). The peptides were generated in a γ‐secretase‐dependent manner, because their abundance was strongly reduced upon γ‐secretase inhibition with DAPT (Fig 2A). The peptides were also specifically derived from sFn14, because they were not detected in the supernatant of HEK293 cells not transfected with the Fn14 plasmid (Fig 2A). The C‐terminal raggedness of the sFn14 peptides is also known for other γ‐secretase substrates, including APP (Steiner *et al*, 2018), and may result from the stepwise cleavage by γ‐secretase and potentially from additional carboxypeptidase‐mediated trimming of an initially generated γ‐secretase cleavage product. The most intensive peptide in the mass range > 7,000 *m*/*z* at 7,294.5 *m*/*z* ended at its C‐terminus in the sequence—PILG (Fig 2A and B), in line with a cleavage within the transmembrane domain (Fig 2C). This peptide was further sequenced at its C‐terminus. Because the intact peptide was too large for sequencing, the peptide was digested with the protease AspN, which cleaves N‐terminally to aspartic acid and generates the C‐terminal peptide DFCLGCAAAPPAPFRLLWPILG (highlighted in bold in Fig 2C), the sequence of which was confirmed by mass spectrometry‐based sequencing (Fig EV1).

The mass spectrometric analysis reveals that Fn14 is cleaved by γ‐secretase within its transmembrane domain, which is in line with the cleavage sites of this protease in other substrates (Güner & Lichtenthaler, 2020).

**Figure 3:**
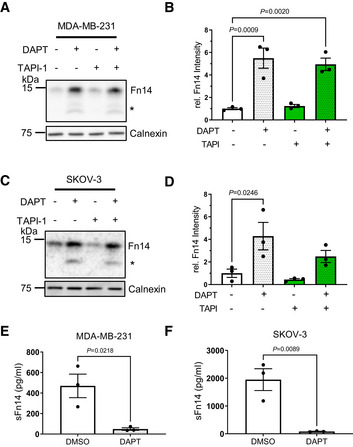
Endogenous Fn14 is processed by protease γ‐secretase Human breast cancer cell line MDA‐MB‐231 shows cellular accumulation of Fn14 upon γ‐secretase inhibition. The cells were treated overnight with γ‐secretase inhibitor DAPT (1 μM), broad‐spectrum metalloprotease inhibitor TAPI‐1 (50 μM), or the corresponding amount of vehicle DMSO as indicated. Lysates were blotted for Fn14 with an antibody that targets the C‐terminal end of the protein, or against calnexin as loading control. The asterisk labels an N‐terminally truncated form of Fn14.Quantification of blots from panel (A). The control condition, where the cells were only treated with vehicle (DMSO), was used as baseline, and its average normalized to 1.Human ovarian cancer cell line SKOV‐3 shows cellular accumulation of Fn14 upon γ‐secretase inhibition. The cells were treated overnight with γ‐secretase inhibitor DAPT (1 μM), broad‐spectrum metalloprotease inhibitor TAPI‐1 (50 μM), or corresponding amount of vehicle DMSO as indicated. Lysates were blotted for Fn14 with an antibody that targets the C‐terminal end of the protein, or against calnexin as loading control. The asterisk labels an N‐terminally truncated form of Fn14.Quantification of blot from panel (C). The control condition, where the cells were only treated with vehicle (DMSO), was used as baseline, and its average normalized to 1.sFn14 is reduced upon γ‐secretase inhibition in MDA‐MB‐231 cells. Conditioned media of the treated cells were collected after overnight DAPT (1 μM) or vehicle treatment. sFn14 concentration was measured by human Fn14 ELISA.sFn14 is reduced upon γ‐secretase inhibition in SKOV‐3 cells. Conditioned media of the treated cells were collected after 48‐h DAPT (1 μM) or vehicle treatment. sFn14 concentration was measured by human Fn14 ELISA. Data Information: All quantification data are shown as mean ± SEM. All the panels have *N* = 3 biological replicates. For panels (B) and (D), the tested conditions were compared against control (DMSO) condition by ordinary one‐way ANOVA and Dunnett's multiple comparison test. For panels (E) and (F), two‐tailed unpaired *t*‐test is used. The *P*‐values that are above 0.05 have not been included into the panels.

### γ‐Secretase cleaves endogenous Fn14 in different tumor cell lines

**Figure EV2:**
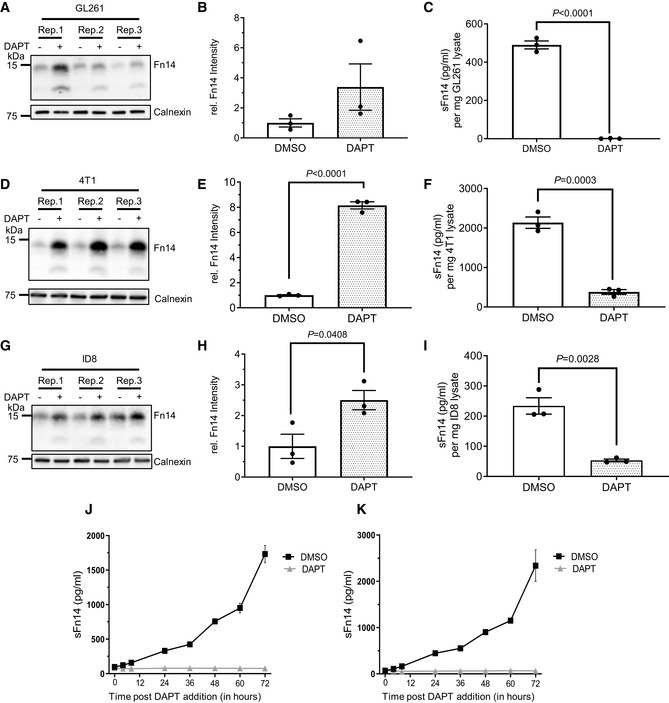
Endogenous Fn14 processing in mouse cell lines by γ‐secretase AMouse glioblastoma cell line GL261 showed cellular accumulation of Fn14 upon γ‐secretase inhibition. Cells were treated overnight with γ‐secretase inhibitor DAPT (1 μM) or vehicle. Lysates of biological replicates (Rep.) were blotted for Fn14 with an antibody that targets the C‐terminal end of the protein, or against calnexin as loading control.BQuantification of blot in panel (A). Intensity values of Fn14 were normalized to the respective Calnexin loading control. The average of the control condition, where the cells were only treated with vehicle (DMSO), was consecutively normalized to 1.CConditioned media of the GL261 cells from panel (A) were collected, and sFn14 levels were measured by ELISA.DMouse breast cancer cell line 4T1 showed cellular accumulation of Fn14 upon γ‐secretase inhibition. Cells were treated overnight with γ‐secretase inhibitor DAPT (1 μM) or vehicle. Lysates were blotted for Fn14 with an antibody that targets the C‐terminal end of the protein, or against calnexin as loading control.EQuantification of blot in panel (D). Intensity values of Fn14 were normalized to the respective calnexin loading control. The average of the control condition, where the cells were only treated with vehicle (DMSO), was consecutively normalized to 1.FConditioned media of the 4T1 cells from panel (D) were collected and sFn14 levels measured by ELISA.GMouse ovarian cancer cell line ID8 showed cellular accumulation of Fn14 upon γ‐secretase inhibition. Cells were treated overnight with γ‐secretase inhibitor DAPT (1 μM) or vehicle. Lysates were blotted for Fn14 with an antibody that targets the C‐terminal end of the protein, or against calnexin as loading control.HQuantification of blot in panel (G). Intensity values of Fn14 were normalized to the respective calnexin loading control. The average of the control condition, where the cells were only treated with vehicle (DMSO), was consecutively normalized to 1.IConditioned media of the ID8 cells from panel (G) were collected and sFn14 levels measured by ELISA.J, KConditioned media of (J) MDA‐MB‐231 or (K) SKOV‐3 cells were collected at indicated time points after DAPT (1 μM) or vehicle treatment and endogenous sFn14 levels were measured by ELISA. Even after 72 h DAPT still completely blocked γ‐secretase, as evidenced by the lack of sFn14 secretion. Data information: All quantification data are shown as mean ± SEM. The *P*‐values that are above 0.05 have not been included into the panels. For all the panels, three biological replicates are performed. For panels (B), (C), (E), (F), (H), and (I), two‐tailed unpaired *t*‐tests were used. For panel (J) and (K), no statistical analysis was performed.

To determine whether γ‐secretase also processes endogenous Fn14, we turned to tumor‐derived cell lines, because Fn14 protein abundance is very low in healthy tissue (Winkles, 2008; Burkly *et al*, 2011). Endogenous Fn14 was detected in the lysate of the human breast cancer cell line MDA‐MB‐231 and the human ovarian cancer cell line SKOV‐3 with an apparent molecular weight of about 14 kDa (Fig 3A–D), which is lower than for the transfected Fn14 constructs because of the lack of the epitope tags. Fn14 was increased upon DAPT treatment as a result of the blocked cleavage by γ‐secretase, while the metalloprotease inhibitor TAPI‐1 had no effect (Fig 3A–D). The soluble product sFn14 was detected by ELISA in the conditioned medium of both cell lines and was strongly suppressed upon inhibition of γ‐secretase with DAPT (Fig 3E and F). Similar results were obtained for three mouse tumor cell lines, glioma GL216, breast cancer 4T1, and ovarian ID8 cells (Fig EV2). DAPT blocked sFn14 efficiently over a time course of 72 h, as determined in MDA‐MB‐231 and SKOV‐3 cells (Fig EV2J and K). We conclude that γ‐secretase cleaves endogenous Fn14.

Interestingly, DAPT treatment did not only increase the abundance of endogenous full‐length Fn14, but also led to the appearance of an approximately 10 kDa band in the cell lysate of all tested cell lines (Figs 3A and C, and EV2A and D, and G, labeled with an asterisk in Fig 3A and C). Since this band was detectable with an antibody to the C‐terminus of Fn14, this band is likely to be N‐terminally truncated compared with full‐length Fn14. Generation of this protein form was not blocked by the metalloprotease inhibitor TAPI‐1 (Fig 3A and C), suggesting that it is either an alternative splice form of Fn14 (Brown *et al*, 2013) induced by the lack of cleavage of full‐length Fn14 upon DAPT treatment or that it is truncated by a protease that is not inhibited by TAPI‐1. A similar situation of alternative cleavages is known for the APP C‐terminal fragment C99, which is efficiently cleaved by γ‐secretase. However, upon γ‐secretase inhibition, C99 is efficiently cleaved in an alternative pathway by the metalloprotease ADAM10, which converts C99 to C83, which lacks the N‐terminal 16 amino acids of C99 (Kuhn *et al*, 2010).

### Inhibition of γ‐secretase cleavage enhances Fn14 signaling

**Figure 4:**
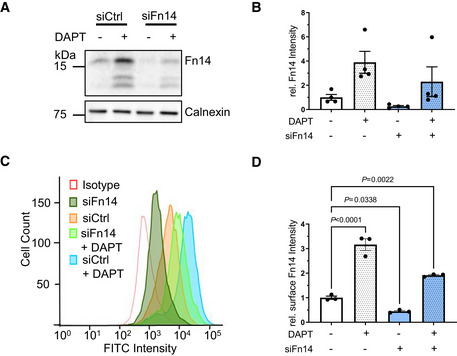
Surface level of endogenous Fn14 is increased upon γ‐secretase inhibition MDA‐MB‐231 cells were transfected with an siRNA pool against human Fn14 or nontargeting control (Ctrl) siRNA. A day after transfection, the cells were treated with γ‐secretase inhibitor DAPT (1 μM) or vehicle overnight. The lysate was collected and blotted against Fn14 (C‐terminal antibody) or calnexin as loading control. Shown are representative blots from *N* = 4 experiments.Quantification of blot in panel (A). The control condition where the cells were only treated with vehicle DMSO and nontargeting siRNA (DMSO + siCtrl) was used as baseline, and its average normalized to 1. *N* = 4 experiments.MDA‐MB‐231 cells were transfected and treated as in panel (A). The treated cells were suspended and labeled with ITEM‐4 antibody that targets an extracellular site of Fn14, or isotype control. Shown are representative histograms from *N* = 3 experiments.The mean intensity of the measurement from panel (C). The control condition where the cells were only treated with vehicle DMSO and nontargeting siRNA (DMSO + siCtrl) was used as baseline, and its average normalized to 1. *N* = 3 experiments. Data information: All quantification data are shown as mean ± SEM. The tested conditions were compared against control (DMSO + siCtrl) condition by ordinary one‐way ANOVA and Dunnett's multiple comparison test. The *P*‐values that are above 0.05 have not been included into the panels. Number of biological replicates performed is indicated in the corresponding panel legend.

The increase in total cellular Fn14 levels after γ‐secretase inhibition with DAPT (Fig 4A and B) also resulted in more Fn14 at the cell surface (3.16 ± 0.24‐fold compared with controls), as seen by flow cytometric analysis of MDA‐MB‐231 cells using antibody ITEM‐4 (Fig 4C and D), which binds to the Fn14 ectodomain. As expected, siRNA‐mediated knockdown of Fn14 reduced surface Fn14 and also the intensity of the Fn14 bands in the cell lysate, as seen by immunoblot (Fig 4A–D). The flow cytometric analysis demonstrates that γ‐secretase controls Fn14 levels at the cell surface.

**Figure 5:**
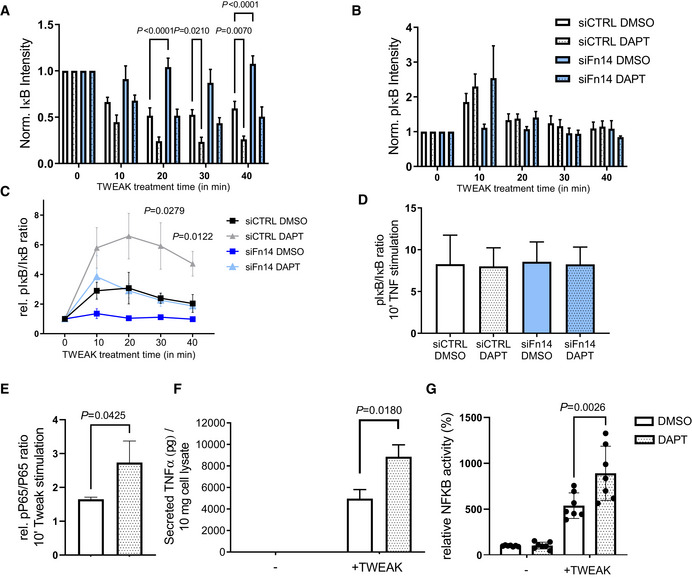
γ‐Secretase inhibition enhances Fn14‐mediated NFκB signaling A, BMDA‐MB‐231 cells were transfected with an siRNA pool against human Fn14 or nontargeting control (Ctrl) siRNA. A day after transfection, cells were treated with γ‐secretase inhibitor DAPT (1 μM) or vehicle overnight. TWEAK (100 ng/ml) was applied for indicated time points. Corresponding immunoblots are in Fig EV3 panel (A) and were used for quantification of (A) IκB and (B) pIκB. The measurements were normalized to the 0 min time point (*N* = 4).CActivation of NFκB is represented as ratio of pIκB to total IκB after TWEAK stimulation (*N* = 4).DNFκB stimulation mediated by TNF is independent of Fn14 and γ‐secretase. MDA‐MB‐231 cells were treated with TNF (10 ng/ml) for 10 min, and the NFκB activation is reported as ratio of pIκB to total IκB (*N* = 4).EMDA‐MB‐231 cells were treated with γ‐secretase inhibitor DAPT (1 μM) or vehicle overnight. TWEAK (100 ng/ml) was applied for 10 min. Quantification of the P65 (NFκB) and pP65 blots in Fig EV3 panel (B). The measurements were normalized to the 0 min time point (*N* = 3).FSKOV‐3 cells were treated with DAPT (1 μM) or vehicle for 48 h and stimulated overnight with TWEAK (100 ng/ml). Conditioned media of the cells were collected, and secreted TNF was measured by ELISA. Shown are data from *N* = 3 experiments.GSKOV‐3 cells were transfected with Luciferase constructs, switched to low serum, treated with DAPT (1 μM) or vehicle overnight with and stimulated with TWEAK (100 ng/ml) for 4 h. Cells were harvested and NFκB reporter activation was measured using a luminometer (*N* = 7). Data information: All quantification data are shown as mean ± SEM. For panels (A) and (B), the tested conditions were compared against control (DMSO + siCtrl) condition of each time point by RM two‐way ANOVA and Dunnett's multiple comparison test. For panel (C), ordinary one‐way ANOVA and Dunnett's multiple comparison test were applied for each time point comparing the tested conditions against control (DMSO + siCtrl). The displayed *P*‐values correspond to the comparison of control with DAPT + siCtrl. For panel (E), a two‐tailed unpaired *t*‐test was performed. For panel (F) and (G), ordinary one‐way ANOVA and Tukey's multiple comparison test were applied. Only the *P*‐values comparing DAPT/DMSO treatment upon stimulation are displayed. Number of biological replicates performed is indicated in the corresponding panel legend. Source data are available online for this figure.

**Figure EV3:**
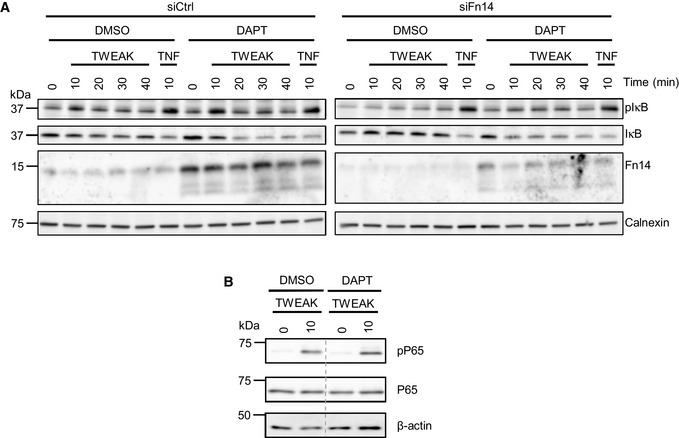
Inhibiting γ‐secretase in MDA‐MB‐231 cells enhances Fn14 mediated NFκB signaling MDA‐MB‐231 cells were transfected with an siRNA pool against human Fn14 or nontargeting control (Ctrl) siRNA. A day after transfection, cells were treated with γ‐secretase inhibitor DAPT (1 μM) or vehicle overnight. Either TWEAK (100 ng/ml) or positive control TNF (10 ng/ml) were applied for indicated time points. The cell lysate was blotted against pIκB and IκB to evaluate NFκB activation or against Fn14 to verify the effect of the DAPT and siFn14 treatment, or against calnexin as a loading control. Shown are representative blots from *N* = 4 experiments.MDA‐MB‐231 cells were treated with γ‐secretase inhibitor DAPT (1 μM) or vehicle overnight. TWEAK (100 ng/ml) was applied for 10 min. The cell lysate was blotted against pP65 and P65 to evaluate NFκB activation or against β‐actin as a loading control. Shown are representative blots from *N* = 3 experiments. The dashed vertical line indicates that sample were run on the same blot but not directly next to each other.

**Figure EV4:**
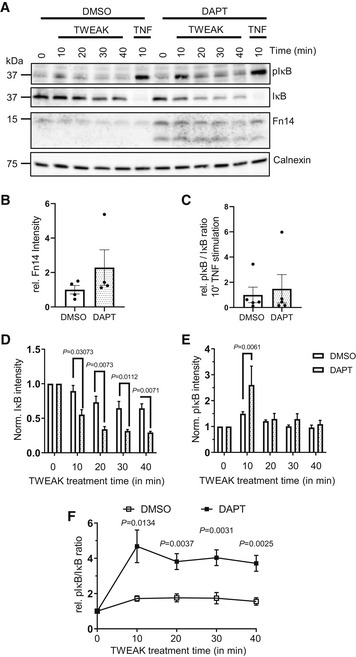
Inhibiting γ‐secretase in glioblastoma cell line U87 enhances Fn14 mediated NFκB signaling AU87 cells were treated with γ‐secretase inhibitor DAPT (1 μM) or vehicle overnight. Either TWEAK (100 ng/ml) or positive control TNF (10 ng/ml) were applied for indicated time points. The cell lysate was blotted against pIκB and IκB to evaluate NFκB activation or against Fn14 to verify the effect of the DAPT and siFn14 treatment, or against calnexin as a loading control. Shown are representative blots from *N* = 4–5 experiments.BU87 cells showed cellular accumulation of Fn14 upon γ‐secretase inhibition. 0 min time point samples of Fn14 blot in panel (A) were quantified, normalized to the respective calnexin loading control and consecutively normalized to vehicle control average. Shown is the Fn14 intensity relative (rel.) to the DMSO control (*N* = 4).Cγ‐Secretase inhibition by DAPT does not alter NFκB stimulation through TNF. U87 cells treated with TNF (10 ng/ml) for 10 min and the NFκB activation reported as ratio of pIκB to total IκB. Shown is the pIκB/IκB ratio relative (rel.) to the DMSO control (*N* = 5).D, EQuantification of the IκB (D) and pIκB (E) blots in panel (A). The measurements were normalized to the 0 min time point. *N* = 5 biological replicates.FThe TWEAK stimulation of Fn14 and activation of NFκB is represented as ratio of pIκB to total IκB, taken from quantifications in (D) and (E). Shown is the pIκB/IκB ratio relative (rel.) to the 0 min time point. *N* = 5 biological replicates. Data information: All quantification data are shown as mean ± SEM. The *P*‐values that are above 0.05 have not been included into the panels. For panel (B) and (C), two‐tailed unpaired *t*‐tests were used. For panels (D) and (E), RM two‐way ANOVAs with Šídák's multiple comparison test have been applied. For panel (F), two‐tailed unpaired *t*‐test have been applied for each time point. For all the panels, the number of biological replicates is reported in the corresponding panel legend.

Because Fn14 is a cell surface receptor, we tested whether γ‐secretase cleavage would also affect Fn14 function and lead to enhanced Fn14 signaling after stimulation with the Fn14‐ligand TWEAK. As a first assay, breast cancer MDA‐MB‐231 cells were treated with TWEAK, which is known to stimulate Fn14 signaling through the NFκB pathway, resulting in phosphorylation and subsequent degradation of the NFκB inhibitor IκB (Burkly, 2014). Using immunoblots, we monitored IκB and phospho‐IκB (pIκB) levels (Fig EV3A) and determined the ratio pIκB/IκB after TWEAK stimulation compared with nonstimulated cells (Fig 5A–C). As a positive control, we treated the MDA‐MB‐231 cells for 10 min with the cytokine TNF, a known activator of the NFκB pathway (Schütze *et al*, 1995). TNF activated the pIκB/IκB ratio eightfold, and this was independent of γ‐secretase inhibition (Fig 5D), because the TNF‐induced signaling pathway is independent of the Fn14 receptor and instead occurs through TNFR1 and TNFR2. TWEAK activated the pIκB/IκB ratio about twofold (Fig 5C). Maximum activation was seen after 10 min and then declined gradually to the 40‐min time point (Fig 5C). Importantly, inhibition of γ‐secretase with DAPT increased the pIκB/IκB ratio more than twofold compared with control‐treated cells. As expected, siRNA‐mediated knockdown of Fn14 reduced the effect size on the pIκB/IκB ratio both in the presence and in the absence of DAPT (Fig 5C). Similar results were obtained for the human glioblastoma cell line U87 (Fig EV4). Tweak stimulation also activated NFκB P65 phosphorylation, as determined in MDA‐MB‐231 cells, and this phosphorylation was increased upon DAPT treatment (Figs 5E and EV3B).

NFκB activation is one of the immediate, proximal signaling events after TWEAK binding to Fn14. To test whether γ‐secretase also controls downstream signaling of Fn14, we used the ovarian cancer SKOV‐3 cell line, which secretes TNF as a result of TWEAK‐stimulated Fn14 signaling in an NFκB‐dependent manner (Vince *et al*, 2008). SKOV‐3 cells show γ‐secretase‐dependent cleavage of Fn14 as demonstrated above (Fig 3C and D). While no TNF was secreted from control‐treated SKOV‐3 cells, TWEAK stimulation led to a robust TNF secretion (Fig 5F). Importantly, TNF secretion was strongly increased by 78% (DMSO: 4970.0 ± 837.5 pg/10 mg lysate DAPT: 8853.2 ± 113.0 pg/10 mg lysate), when γ‐secretase was inhibited with DAPT. As an additional readout for downstream signaling, we transfected SKOV‐3 cells with an established reporter construct expressing luciferase under the control of a NFκB‐responsive promoter. Addition of TWEAK‐stimulated luciferase expression and this increase was enhanced by 60% upon DAPT treatment (Fig 5G).

Taken together, these experiments demonstrate that γ‐secretase affects Fn14 signaling through the NFκB pathway.

### Fn14 is processed by γ‐secretase *ex vivo*

**Figure 6:**
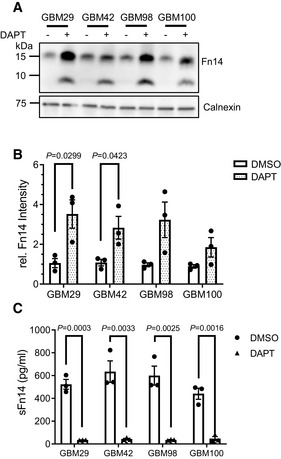
γ‐Secretase mediates proteolysis of Fn14 in primary cells from human glioblastoma biopsy samples Cellular Fn14 in *ex vivo* glioblastoma samples increased upon γ‐secretase inhibition. Primary cells from four different glioblastomas were treated with DAPT (1 μM) or vehicle overnight. Lysates were blotted against Fn14 and calnexin as loading control.Quantification of the blot in panel (A). For each glioblastoma, the relative (rel.) mean intensity of the normalized Fn14 vehicle condition was used for normalization.sFn14 was strongly reduced upon γ‐secretase inhibition in primary GBM cells. Conditioned media of the treated cells from panel (A) were collected after overnight DAPT (1 μM) or vehicle treatment. sFn14 levels in these samples were measured by human Fn14 ELISA. Data information: All quantification data are shown as mean ± SEM. The tested conditions were compared against their corresponding control (DMSO) condition using two‐tailed unpaired *t*‐tests. The *P*‐values that are above 0.05 have not been included into the panels. For all the panels, three biological replicates were performed. Source data are available online for this figure.

To determine whether γ‐secretase‐mediated Fn14 proteolysis also occurs in primary human cells *ex vivo*, we analyzed primary cells obtained from tumor tissue of four different patients with glioblastoma (GBM) (Dong *et al*, 2015), where Fn14 expression is linked to poor patient survival (Tran *et al*, 2006; Perez *et al*, 2016; Hersh *et al*, 2018). Similar to the human and murine cell lines investigated above, DAPT increased Fn14 protein in the cell lysate and nearly completely abolished sFn14 release into the conditioned medium (Fig 6A–C), demonstrating γ‐secretase‐dependent sFn14 release from primary human tumor cells. Similar to the cell lines (Fig 3A and C), DAPT treatment also induced generation of the alternative, lower‐molecular weight form of Fn14 (Fig 6A), which may be an alternative splice form of Fn14 (Brown *et al*, 2013) or a proteolytically truncated form.

### γ‐Secretase‐dependent sFn14 release is detected in mouse and human blood

**Figure 7:**
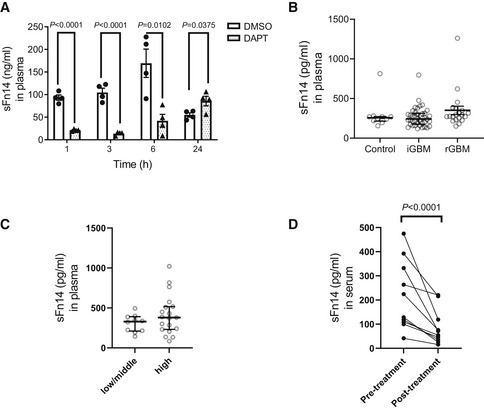
*In vivo* sFn14 is generated by γ‐secretase activity Mice were treated with single dose of 100 mg/kg DAPT (or vehicle DMSO) and their plasma was collected at the indicated time points after dosing. Individual mice were used at each time point. sFn14 levels in these samples are displayed (*N* = 4).Plasma samples from 10 controls and GBM patients with an initial diagnosis of GBM (iGBM, 40 patients) or a recurrent GBM (rGBM, 20 patients) were collected and sFn14 levels were measured by ELISA.Plasma samples from 10 ovarian cancer patients with low/middle and 19 patients with high tumor burden were collected and sFn14 levels were measured by ELISA. The tumor burden was clinically determined by computer tomographic scans.Serum samples from 10 patients with refractory multiple myeloma were collected pre‐ and posttreatment with 25 mg LY3039478 administered in three daily doses over 5 days. sFn14 levels in these samples are displayed (*N* = 10). Data information: For panel (A), data are shown as mean ± SEM. Different mice were used for each time point. Two‐tailed unpaired t‐test was applied. For panel (B) and (C), median of the measurements, along with interquartile range borders, is displayed. In panel (B), ordinary one‐way ANOVA with Tukey's multiple comparison test was applied, but no value passed our *P*‐value display threshold of 0.05. For panel (C), a two‐tailed unpaired *t*‐test was applied, but no value passed our *P*‐value display threshold of 0.05. For panel (D), ratio‐paired *t*‐test was used to compare the sFn14 levels pre‐ and posttreatment. For all the panels, the number of biological replicates is reported in the corresponding panel legend.

Fn14 was previously detected in serum and urine of a mouse model of the kidney disease nephrotoxic nephritis and in serum of human healthy volunteers (Sharif *et al*, 2016; Chen *et al*, 2018, 2019), but it remained unclear whether this form of Fn14 corresponds to the proteolytically generated sFn14 or instead represents full‐length Fn14, potentially released from multivesicular bodies or dead cells. To determine whether the γ‐secretase‐generated sFn14 is indeed detectable in plasma, we treated mice with a single dose i.p. of either DAPT or DMSO as vehicle and collected plasma 1, 3, 6, and 24 after dosing. While ELISA‐measured sFn14 concentration in control‐treated animals varied with time, potentially due to the diurnal rhythm, DAPT reduced plasma sFn14 by about 80% already 1 h after dosing (Fig 7A). The reduction was maintained at 3 and 6 h after dosing and increased to the levels of the control‐treated mice at the 24‐h time point, consistent with the *in vivo* half‐life of DAPT (Dovey *et al*, 2001).

From the mouse plasma analysis, we conclude that sFn14 is a regular mouse plasma constituent and released by γ‐secretase *in vivo*, demonstrating the physiological relevance of sFn14 release.

Next, we tested whether sFn14 is also detectable in human blood by ELISA and how it differs among healthy individuals and among individuals with GBM, where Fn14 shows increased expression (Tran *et al*, 2006; Perez *et al*, 2016; Hersh *et al*, 2018). In healthy control individuals, plasma sFn14 concentrations were in a range from 156 to 280 pg/ml with only one individual showing an approximately threefold increased sFn14 concentration (814 pg/ml) (Fig 7B). In the patient groups with an initial GBM diagnosis (iGBM) or with a recurrent GBM (rGBM), the sFn14 concentration was in a similar range but showed a trend to an increase in the rGBM group.

Fn14 is also linked to ovarian cancer (Gu *et al*, 2013) and sFn14 was detected in plasma of ovarian cancer patients (Fig 7C). sFn14 concentration did not differ between patients with a low/middle versus high tumor burden as determined by computer tomography, but showed a larger spread in the high tumor burden group. These results indicate that the sFn14 plasma concentration may depend on tumor progression, but larger patient numbers and in particular longitudinal samples are required to evaluate the potential of sFn14 as a tumor staging biomarker.

Because sFn14 is generated by γ‐secretase and detected in mouse and human blood, we tested the possibility to use sFn14 as serum‐based treatment‐response biomarker for γ‐secretase inhibition in clinical trials. Serum samples were obtained from 10 patients with refractory multiple myeloma enrolled on a clinical trial (NCT03502577) of chimeric antigen receptor modified T cells (CAR T) targeting BCMA (Pont *et al*, 2019). Prior to CAR T‐cell infusion, patients were treated with three oral doses (25 mg each) of the γ‐secretase inhibitor LY3039478 over 5 days to increase surface BCMA levels and enhance efficacy of the CAR T‐cell treatment. sFn14 was measured in the serum prior to the first dose and 4–6 h after the third dose of γ‐secretase inhibitor. As a result of γ‐secretase inhibition, sFn14 concentration was reduced in all individuals, reaching up to 80% inhibition (Fig 7D).

We conclude that a soluble form of Fn14 is detectable in human plasma and serum, released in a γ‐secretase‐dependent manner and is suitable as a pharmacodynamic marker for γ‐secretase activity *in vivo*.

## Discussion

**Figure 8:**
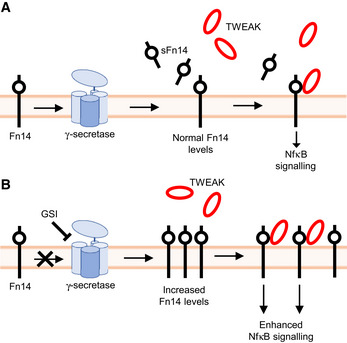
Illustration of how γ‐secretase controls abundance and signaling of Fn14 and release of sFn14 Under normal conditions, Fn14 is processed by γ‐secretase, which generates sFn14 and degrades Fn14. Fn14 ligand TWEAK binds to remaining Fn14 on the surface, which activates downstream NFκB signaling.Upon inhibition of γ‐secretase activity, Fn14 proteolysis is reduced. This ablates sFn14 release and increases Fn14 in the cells and on the cell surface, allowing enhanced Fn14‐mediated signaling upon TWEAK ligand binding.

Fn14 is a cell surface signaling receptor with key functions in tissue homeostasis and tissue injury. Fn14 is also linked to numerous chronic diseases, where it is used as a target for drug development. Despite its physiological and medical role, relatively little is known about how the function and signaling of Fn14 are controlled at the cellular level. We report that the intramembrane protease γ‐secretase is a new modulator of Fn14 signaling and establish Fn14 as a novel, but unusual substrate for γ‐secretase. This protease cleaves Fn14 within its transmembrane domain and provides a mechanism for controlling the abundance of Fn14 at the cell surface and for attenuating its function in signal transduction (Fig 8). Moreover, we demonstrate that the cleaved soluble Fn14 is detectable *in vivo* in plasma and may be useful as an easily accessible pharmacodynamic activity marker of γ‐secretase.

γ‐Secretase has fundamental functions in physiology, for example for Notch receptor signaling during development (De Strooper *et al*, 1999), but also contributes to diseases, such as Alzheimer's disease (AD) and various tumors, where it is tested as a drug target (De Strooper, 2014; McCaw *et al*, 2020). Mechanistically, γ‐secretase has a broad sequence specificity and cleaves around 150 membrane proteins within their single transmembrane domain (Güner & Lichtenthaler, 2020). All of them have type I‐oriented transmembrane domains, that is they have their N‐terminus in the luminal or extracellular domain and the C‐terminus located in the cytosol, and this is also true for Fn14. As shown for APP, γ‐secretase typically cleaves a substrate multiple times, starting with a first cleavage at the cytosolic end of the substrate's transmembrane domain, followed by several consecutive cleavages toward the middle and N‐terminal part of the transmembrane domain until the remaining transmembrane domain is short enough to slip out of the membrane, leading to secretion of the substrate's remaining ectodomain (Takami *et al*, 2009). Presumably, the consecutive cleavages are the reason for why the C‐terminus of sFn14 shows heterogeneity.

Although Fn14 shares several features of a typical γ‐secretase substrate—such as type I orientation and multiple cleavages within its transmembrane domain—Fn14 is distinct from typical γ‐secretase substrates. Known γ‐secretase substrates generally have long extracellular domains and can only be cleaved by γ‐secretase after the ectodomain has been truncated by other proteases, such as ADAMs and BACE1 (Struhl & Adachi, 2000; Lichtenthaler *et al*, 2018), which prevents unwanted cleavage of the full‐length protein by γ‐secretase. Mechanistically, the substrate length is controlled by the γ‐secretase subunit nicastrin, which forms a lid on the extracellular side of the active site of γ‐secretase (Bai *et al*, 2015; Bolduc *et al*, 2016) and accepts only substrates with shortened ectodomains. Fn14 is an unusual substrate in that it has an extracellular domain of only 53 amino acids, which appears short enough to allow direct cleavage by γ‐secretase without the need of prior ectodomain truncation. Only one other γ‐secretase substrate, the receptor B‐cell maturation antigen (BCMA), is known to also have a short extracellular domain (54 amino acids) and was validated to be cleaved by γ‐secretase without prior truncation (Laurent *et al*, 2015). Additionally, a very recent study suggested that synaptotagmin 7 (Syt7), which acts as a calcium sensor in membrane trafficking and has an extracellular domain of only 16 amino acids, may also be cleaved by γ‐secretase (Vevea *et al*, 2021). We propose that Fn14, BCMA, and Syt7 are the founding members of a new class of γ‐secretase substrates, which we refer to as naturally short γ‐secretase substrates.

Fn14 has fundamental roles in healthy and diseased tissues, but little is known about the mechanisms that control Fn14 function. Upregulation of Fn14 during tissue stress or injury was assumed to be the main mechanism controlling Fn14 signaling (Winkles, 2008; Burkly *et al*, 2011; Poveda *et al*, 2021), but our study demonstrates that γ‐secretase‐mediated shedding of Fn14 is a new and additional mechanism to control Fn14 signaling and its surface abundance. Surface Fn14 is used as a target for the delivery of immunotoxins and antibody‐drug conjugates to reduce the number of Fn14‐expressing tumor cells (Zhou *et al*, 2013, 2014; Lerchen *et al*, 2018; Alvarez de Cienfuegos *et al*, 2020; Dancy *et al*, 2020). We propose that short‐term addition of a γ‐secretase inhibitor (GSI) may be an excellent way to enhance therapeutic efficacy of Fn14‐targeted immunotoxins through two mechanisms, (i) an increase of surface Fn14 levels available for immunotoxin binding and (ii) a reduction in sFn14 which can act as a decoy receptor that sequesters Fn14‐targeted drugs (Yepes *et al*, 2005). Feasibility of such an approach was recently shown using another γ‐secretase substrate, BCMA, for treatment of multiple myeloma with chimeric antigen receptor (CAR) T cells in a mouse model and is currently tested in a clinical trial for multiple myeloma, again using CAR T‐cell therapy (Pont *et al*, 2019). An application of GSIs to enhance surface Fn14 levels may also be considered when agonistic Fn14 antibodies, such as BIIB039, are used to stimulate antiproliferative signaling of Fn14 to reduce tumor growth, for example in xenograft models of colon cancer and gastric tumors (Michaelson *et al*, 2011). However, in other disease conditions, increased Fn14 signaling may be detrimental so that the use of a GSI may enhance the underlying disease process. One example is tumor‐induced cachexia, which was prevented by antagonistic Fn14 antibodies and prolonged survival in a mouse model with a xenograft of Fn14‐overexpressing mouse embryonic fibroblasts (Johnston *et al*, 2015).

The γ‐secretase cleavage product sFn14 may serve as an excellent and easily measurable pharmacodynamic blood biomarker for γ‐secretase activity *in vivo*, particularly in clinical trials of cancer with GSIs. High‐dose or prolonged GSI treatment is associated with significant toxicity, such as in the gastrointestinal tract, presumably resulting from inhibition of numerous γ‐secretase substrates, including Notch (Moore *et al*, 2020). Thus, γ‐secretase inhibition needs to be closely monitored to control side effects and determine target engagement, which, however, is still not routinely feasible. One γ‐secretase cleavage product is the AD‐related Aβ peptide, which is readily detectable in the brain cerebrospinal fluid (CSF) where it is typically monitored in AD trials. Another γ‐secretase cleavage product detectable in CSF is the APLP1‐derived peptide APL1β (Yanagida *et al*, 2009). Both Aβ and APL1β in CSF respond to γ‐secretase inhibition. Yet, the Aβ and APL1β concentrations in plasma are below the detection limit or require substantial plasma fractionation or immunoprecipitation for analysis. Another approach to measure γ‐secretase activity may be positron emission tomography (PET), for which a first γ‐secretase‐targeting PET ligand has recently been developed (Nie *et al*, 2021). However, CSF collection and PET are invasive, expensive, and complex procedures not suitable for routine diagnostics. Our study reveals that sFn14 is easily measurable in plasma of both mice and humans, responds well to γ‐secretase inhibition and may be considered for measuring treatment responses to stratify patients or for a precision medicine approach in future clinical trials with GSIs for different diseases, ranging from solid tumors to AD. Additionally, sFn14 may help to develop substrate‐preferring GSIs which preferentially block generation of the Aβ peptide in AD over the cleavage of other substrates, such as Fn14, and, thus, avoid the side effects observed upon chronic GSI treatment. Measurement of sBCMA in plasma is now also feasible (Pont *et al*, 2019) and may potentially be combined with sFn14 measurement.

Taken together, this study demonstrates a new concept for Fn14 signaling, namely its control through γ‐secretase‐mediated Fn14 proteolysis, which may be exploited for therapeutic approaches. Additionally, this work establishes a new class of γ‐secretase substrates, referred to as naturally short γ‐secretase substrates, which can be instrumental in better understanding the mechanisms of substrate recognition and substrate cleavage through γ‐secretase and thereby help to develop safer drugs targeting γ‐secretase. Finally, this work reveals that sFn14 can be routinely detected in blood and may serve as an activity marker for γ‐secretase.

## Materials and Methods

### Overexpression of Fn14 in cell culture and Western blots

Human embryonic kidney 293 EBNA (HEK293E) cells were seeded on precoated 24‐well plates (Corning) and transfected with a pcDNA3.1 plasmid carrying human Fn14 cDNA tagged with an N‐terminal HA‐tag (YPYDVPDYA) after the Fn14 signal peptide and a C‐terminal double FLAG tag with a three amino acid linker (GLEDYKDDDDKDYKDDDDK). As control, C99 (99 amino acid long C‐terminal part of APP that corresponds to the β‐secretase‐cleaved C‐terminal fragment) cDNA carrying the same epitope tags was used. Transfection was performed by Lipofectamine™ 2000, according to the manufacturer's instructions (Thermo Fisher, US). Cells were incubated overnight in 500 μl DMEM (Gibco) with 10% FCS, containing either 1 μM of the γ‐secretase inhibitor DAPT (Sigma), 50 μM of the broad‐spectrum metalloprotease inhibitor TAPI‐1 (MilliporeSigma), or DMSO as control. Conditioned media were collected and centrifuged for 1 h at 100,000 *g* and stored at −20°C. Cells were lysed with 300 μL STET (150 mM NaCl, 50 mM Tris [pH 7.5], 2 mM EDTA, 1% Triton X‐100) buffer containing 1× protease inhibitor (Sigma). The lysate and the correspondingly adjusted volume of conditioned media were loaded on Schägger gradient gels (Schagger, 2006). Detection of overexpressed proteins and their proteolytic products was made with anti‐HA.11 (Covance, clone 16B12, Cat MMS‐101P) and anti‐FLAG (Sigma‐Aldrich, clone M2, Cat F1804) antibodies.

PS1 KO and PS1/2 dKO have been generated from HEK293 cells that stably overexpress APP containing Swedish mutation (Tagami *et al*, 2017). These cells were transfected with human Fn14 cDNA as described above. Conditioned media collection, cell lysis, and immunoblotting for these cells were identical with HEK293E. Additionally, the following antibodies were used in the blotting; mouse anti‐PS1‐CTF (Merck Millipore, clone PS1‐loop, Cat MAB5232), mouse C‐terminal anti‐APP (Colombo *et al*, 2013) which detects both C‐terminal fragments (including C83, which was analyzed here) and APP and rabbit anti‐calnexin (Enzo, Cat ADI‐SPA‐860). Note that calnexin runs on the Tris Tricine Schägger gels at 75 kDa, instead of 100 kDa.

### Isolated membrane incubation assay to measure γ‐secretase activity

The assay performed as previously described (Sastre *et al*, 2001). Shortly, HEK293 cells were transfected with the epitope‐tagged Fn14 plasmid. The membranes were isolated and incubated in reaction buffer (150 mM sodium citrate pH 6.4 with 1× protease inhibitor), in the presence of either 1 μM DAPT or vehicle. The reaction was incubated at the indicated temperatures and times. At the end of the reaction, samples were boiled in Laemmli buffer, loaded on Schägger gels and blotted.

### MALDI

To obtain the sFn14 peptide samples, HEK293 cells were seeded into 100 mm cell culture plates. Next day, the cells were transfected with tagged Fn14 as described above. The transfected cells were treated with DAPT (1 μM), or DMSO vehicle overnight. The conditioned media were collected and centrifuged for 1 h at 100,000 *g*. Supernatant was collected, and HA‐antibody conjugated beads (Sigma) were used for immunoprecipitating HA‐tagged sFn14. The peptide on the beads were eluted with 0.3% TFA and 50% ACN in dH_2_O.

Matrix‐assisted laser desorption ionization mass spectrometry (MALDI‐MS) was performed on the eluates to assess the peptide composition. 1 μl of each sample (*n* = 4) was mixed with 1 μl of saturated Sinapinic acid (SA) solution in 50% ACN and 0.2% TFA and spotted on a MTP 384 ground steel target plate (Bruker Daltonik GmbH, Germany). Masses of the peptides were measured in a rapifleX MALDI Tissuetyper MALDI‐TOF/TOF mass spectrometer (Bruker Daltonik GmbH) equipped with a SmartBeam 3G laser. Spots were measured in a positive linear mode using a mass range of 3,000–16,000 Da and high Realtime Smoothing (20 MHz). Ensuing spectra were imported into mMass (Strohalm *et al*, 2008, 2010), baseline subtracted and peaks (with S/N > 2) were picked. The observed masses were then further compared with the expected theoretical masses (Fig 2B).

To confirm the identities of the peptides, we proceeded to perform a fragment ion analysis (MS/MS) of selected masses (precursor ions). However, since the masses detected in the abovementioned experiment(s) were beyond the range that could be fragmented by the instrument, we further proteolytically digested the peptides. Immunoprecipitated sFn14 samples were neutralized with 50 mM ammonium bicarbonate. The peptides were reduced and alkylated using DTT and iodoacetamide, respectively. Subsequent digestion was performed by the protease AspN by single‐pot, solid‐phase‐enhanced sample preparation (SP3) (Hughes *et al*, 2018). The resulting peptides were dried in a vacuum centrifuge, and resuspended in the elution solution described above. 1 μl of sample was mixed with 1 μl of saturated α‐Cyano‐4‐hydroxycinnamic acid (HCCA) solution in 70% ACN and 0.1% TFA, spotted on MTP 384 ground steel target plate (Bruker Daltonik GmbH) and measured in rapifleX MALDI Tissuetyper MALDI‐TOF/TOF mass spectrometer (Bruker Daltonik GmbH). Spots were measured in a positive reflector mode using a mass range of 700–3,200 Da.

Fragment ions were measured in the MS/MS mode using a mass range of 60–2,500 Da. and an isolation window of ±10 Da. 4,000 fragment shots with a laser power boost of 70% were used to measure the fragment ions. Sum spectra (from at least 12,000 shots) were acquired for each sample and further analyzed in FlexAnalysis (Bruker Daltonik GmbH). The fragment ion mass lists were compared (within ± 1 Da. tolerance) with theoretically generated mass lists of the peptide DFCLGCAAAPPAPFRLLWPILG at http://db.systemsbiology.net:8080/proteomicsToolkit/FragIonServlet.html. We used a charge state of +1 and a fixed addition of 57 Da to each Cys residue to account for the carbamidomethyl modification arising due to the alkylation reaction. Since the fragmentation mode in rapifleX is not defined systematically, all possible types of fragment ions were considered during the analysis (Fig EV1).

### Culture of cell lines and primary cells

For maintaining the cell lines HEK293E, MDA‐MB‐231, and U87 cells, DMEM (Gibco) supplemented with 10% FCS and 1% penicillin/streptomycin were used as the culture media. For GL261 cell line, additionally, 1% non‐essential amino acids were added to the culture media. SKOV‐3 cells were maintained in McCoy's 5A media (Gibco) supplemented with 10% FCS and 1% penicillin/streptomycin. 4T1 cells were maintained in RPMI 1640 media (Gibco) with 10% FCS and 10 mM HEPES. The cell lines that have been subjected to NFκB activation, namely MDA‐MB‐231 and U87 cells, were starved with 1% FCS containing media one day prior to the experiment. ID8 cells were maintained in DMEM media (Gibco) supplemented with 5% FCS, 10 mM Hepes and 1% ITS solution containing 10 μg/ml insulin, 5.5 μg/ml transferrin and 6.7 ng/ml sodium selenite. Cells were regularly tested to be mycoplasma‐free.

### Flow cytometry

MDA‐MB‐231 cells were seeded in 12‐well plates. The cells were transfected with siRNA pool (ON‐TARGETplus Human TNFRSF12A (51330) siRNA ‐ SMARTpool, Dharmacon) against Fn14 or with non‐targeting siRNA, using Lipofectamie RNAiMAX reagent and following producer's protocol. The following day, the media of the cells were switched to 1% FCS media, containing either 1 μM DAPT or DMSO. After overnight incubation, the cells were suspended with EDTA treatment, and incubated with N‐terminal Fn14 antibody (eBioscience, clone ITEM‐4, Cat 14‐9018‐82), which targets the extracellular domain of Fn14, or an isotype‐matching antibody targeting a cytosolic protein have been used (anti‐OXSR1, clone 2A5, Cat H000009943). As the secondary antibody, Alexa Fluor anti‐mouse 488 (Thermo, Cat A21202) was used. The stained cells, and the control cells, were measured by flow cytometry with a band pass filter of (527 ± 32) nm (BD FACS Melody), and analyzed by FlowJo v10.4.1 software (BD Life Sciences). For each sample, 100,000 events were acquired and debris as well as cell aggregates were excluded based on forward and side scatter plots.

### NFκB activation assay

For MDA‐MB‐231 cells, the cell seeding and the treatments were carried out as indicated above. Shortly, the cells were transfected with siRNAs (siFn14 or siCtrl, Dharmacon) and treated with DAPT (or DMSO) in 1% FCS media 1 day before the experiment. The treatment with 100 ng/ml human recombinant TWEAK (Biotechne) or 10 ng/ml human recombinant TNF (Biotechne) were carried out in the indicated time points. The cells were lysed with RIPA buffer (150 mM NaCl, 10 mM Tris–HCl pH 8.0, 2 mM EDTA, 1% Triton‐X100, 0.1% sodium deoxycholate, 0.1% SDS) containing protease inhibitor and phosphatase inhibitor PhosStop (Roche). Lysates were loaded on Schägger gels or 12% SDS‐gel and immunoblotted with the following antibodies: rabbit anti‐Fn14 (Cell Signaling, Cat #4403), rabbit anti‐IκBa (Cell Signaling, Cat #9242), mouse anti‐phospho(Ser32/36) IκBa (Cell Signaling, clone 5A5, Cat #9246), rabbit anti‐P65 (Cell signaling, clone D14E12, Cat #8242), rabbit anti‐phospho P65 (Cell Signaling, clone 93H1, Cat # 3033), mouse anti‐β‐actin (Sigma, Cat A5316), and rabbit anti‐calnexin (Enzo, Cat ADI‐SPA‐860).

### NFκB luciferase assay

SKOV‐3 cells were seeded and maintained as described above. One day after seeding, cells were switched to FBS low medium (1%) and transfected with an NFκB reporter luciferase plasmid (Laurent *et al*, 2015) and pRenilla TK (Promega). Cells were treated overnight with DAPT (or control) and stimulated with TWEAK the next morning. After 4 h, cells were harvested and then lysed according to the manufacturer's instructions using the Dual‐Glo^®^ Luciferase Assay System (Promega). Samples were analyzed in duplicates using CLARIOstar Plus (BMG Labtech).

### Fn14 and TNF ELISAs

For all the tested cell lines, the treatments with the DAPT (or control) were performed overnight, expect for SKOV‐3 cells, which were subjected to the treatment for 48 h, or in accordance with the indicated time points. For the DAPT time course experiment, cells were pretreated for 1 h with DAPT or vehicle, medium was switched and fresh DAPT or DMSO was added for time point 0. The following protocol is applied for the cell lines tested. The conditioned media from the cells were collected, and centrifuged to get rid of cellular debris. For the human serum samples, cell lines and also for the patient‐derived glioblastoma cells, the collected samples were analyzed with human TNFRSF12A/TWEAKR ELISA Kit PicoKine™ (Boster). For the mouse serum samples and cell lines, the analysis performed by RayBio® Mouse TWEAK R (TNFRSF12) ELISA Kit (RayBiotech). The conditioned media from SKOV‐3 cells were tested for secreted TNF. The measurement was taken via V‐PLEX human TNF kit (MSD), following the kit protocol.

### Patient‐derived glioblastoma cells

Human GBM primary cells were prepared from WHO grade IV GBM patients as described previously (Dong *et al*, 2015; Hannen *et al*, 2019). Briefly, tumor tissues were collected immediately after surgery. Tissues were washed in HEPES buffer, mechanically homogenized and digested with Trypsin/ EDTA solution at 37°C. Tissue fragments were then passed over an 80 μm cell strainer and centrifuged at 200 g for 5 min. Cells were seeded after two additional washes with PBS. All steps performed as described previously (Dong *et al*, 2015). For GBM cell protein extraction, 4 × 10^5^ cells were seeded in a six‐well plates overnight, changed with fresh medium containing 1 μM DAPT and incubated overnight. Cells were washed three times with cold PBS. Conditioned medium was used for ELISA measurements (described above). Total protein extracts were generated with RIPA buffer including a complete protease inhibitor cocktail (Thermo Scientific) and phosphatase inhibitor (Thermo Scientific).

### Treatment of mice with DAPT

Approval for all experimental animal procedures was obtained from the Government of Upper Bavaria (ROB‐55.2‐2532.Vet_02‐17‐186). The treatment and handling of animals was carried out in accordance with the institutional guidelines of the Preclinical Research Center at the Technical University of Munich. The animal facility is designed following the EU guidline 2010/63 for animal housing, including humidity of 45–60%, adjusted room temperature between 20 and 24°C and a 12‐h day and night cycle with twilight phases. The facilities are classified as SPF. Food and water were sterilized and available *ad libitum*. The animals had an adjustment period of at least 1 week before any experiments were performed. Eight‐week‐old Balb/c mice were obtained from Charles River. *In vivo* experiments were performed with four mice per group. The animals were treated with a single dose of DAPT (100 mg/kg in 100 μl DMSO) or DMSO only via i.p. injection. Blood samples were acquired via punctuation of the vena facialis 1, 3, 6, and 24 h after treatment. Blood plasma was generated via citrate anticoagulation in a 1:9 ratio followed by centrifugation at 10,000 *g* for 10 min. Samples were immediately stored at −80°C until further usage.

### Plasma samples from healthy donors and GBM patients

For work on patient materials (tumor tissue and plasma samples), ethical approval was obtained from the local ethics committee at Medical Faculty, Marburg University, file number 185/11. Informed written consent was obtained from every patient included in this study. Tumor diagnosis was confirmed by a certified neuropathologist (Marburg University).

### Plasma samples from ovarian cancer patients

For the analysis of patient‐derived plasma, ovarian cancer patients who were treated between 2019 and 2021 at the Technical University of Munich Hospital (Klinikum rechts der Isar, Department of Obstetrics and Gynecology) were included in our study. Blood samples were acquired at time of the diagnosis. Plasma was collected after anticoagulation with EDTA for 30 min, followed by centrifugation at 10,000 *g* for 10 min at 4°C. Samples were stored in liquid nitrogen for further use. Written informed consent was obtained from all patients. The study was approved by the institutional reviewer board of the Technical University of Munich (approval 566/19S) in accordance with the Declaration of Helsinki and the Department of Health and Human Services Belmont Report.

### Serum samples from multiple myeloma patients

Serum samples were obtained from patients with refractory multiple myeloma enrolled on a phase 1 clinical trial (NCT03502577) evaluating BCMA CAR T cells combined with a γ‐secretase inhibitor. The study was conducted in accordance with the principles of the Declaration of Helsinki and the Department of Health and Human Services Belmont Report and with the approval of the Fred Hutchinson Cancer Research Center Institutional Review Board.

### Statistics

The statistical tests used in the analyses are indicated within the respective figure legend. The statistics were calculated by GraphPad 9 software. Samples were not blinded before analysis, but samples were measured in an automated fashion where possible to avoid bias. Data are presented from biological replicates.

## Author contributions

**Gökhan Güner:** Conceptualization; investigation; writing – review and editing. **Marlene Aßfalg:** Investigation; methodology; formal analysis; writing – review and editing. **Kai Zhao:** Investigation; writing – review and editing. **Tobias Dreyer:** Investigation; writing – review and editing. **Shibojyoti Lahiri:** Investigation; methodology. **Yun Lo:** Investigation. **Biana Ionela Slivinschi:** Investigation. **Axel Imhof:** Formal analysis; investigation. **Georg Jocher:** Investigation; methodology. **Laura Strohm:** Methodology. **Christian Behrends:** Methodology; writing – review and editing. **Dieter Langosch:** Conceptualization; writing – review and editing. **Holger Bronger:** Investigation. **Christopher Nimsky:** Formal analysis; supervision. **Joerg W Bartsch:** Investigation; writing – review and editing. **Stanley R Riddell:** Funding acquisition; investigation; writing – review and editing. **Harald Steiner:** Conceptualization; writing – review and editing. **Stefan F Lichtenthaler:** Conceptualization; formal analysis; supervision; funding acquisition; writing – original draft; writing – review and editing.

In addition to the CRediT author contributions listed above, the contributions in detail are:

GG and SFL designed the study with help from DL and HS; GG conducted most experiments with help from BIS and MA; SL and AI conducted mass spectrometric experiment; mouse experiment was carried out by TD and HB; GJ, LS and CB helped with flow cytometry analysis; GBM samples were collected and provided by KZ, JWB, CN; multiple myeloma samples were analyzed by SR and YL; data interpretation was done by GG, SL, DL, HS, SFL; manuscript was written by SFL with input from all authors.

## Disclosure and competing interests statement

SRR is a founder of Juno Therapeutics, now Bristol Myers Squibb and Lyell Immunopharma and serves as an advisor to Juno Therapeutics, Lyell Immunopharma, and Adaptive Biotechnologies. All other authors declare no conflict of interest.

**For more information**

- Alzheimer's Association information on Alzheimer's disease: https://www.alz.org/
- Uniprot Entry on human Fn14: https://www.uniprot.org/uniprotkb/Q9NP84/entry
- Uniprot Entry on murine Fn14: https://www.uniprot.org/uniprotkb/Q9CR75/entry

## Supporting information



Expanded View Figures PDF

PDF+

Source Data for Figure 5

Source Data for Figure 6

## Data Availability

This study includes no data deposited in external repositories.
