# Supplementary material for: Proteolytically generated soluble Tweak Receptor Fn14 is a blood biomarker for γ‐secretase activity
Source: EMBO Mol Med. 2022 Sep 7;14(10):e16084. doi: 10.15252/emmm.202216084 (PMC9549706; doi:10.15252/emmm.202216084)
Supplement: Supplementary file 1 — Expanded View Figures PDF [file EMMM-14-e16084-s004.pdf]

Expanded View Figures

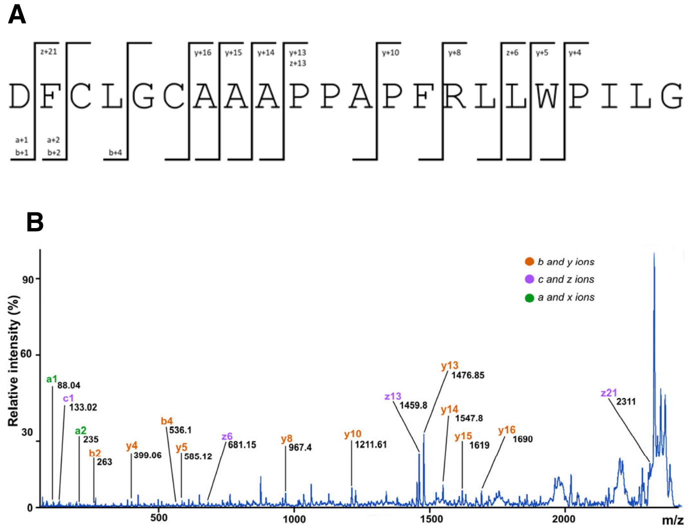

Figure EV1. Fragmentation spectrum and sequencing of C-terminal sFn14 peptide.

A sFn14, immunoprecipitated from the conditioned medium of HA-Fn14-trasfected HEK293E cells, was digested with AspN. The peptide corresponding to the C-terminal fragment with the sequence "DFCLGCAAAPPAPFRLLWPILG" was fragmented and sequenced. Detected fragment ions are schematically indicated.

B Fragmentation spectrum of the peptide "DFCLGCAAAPPAPFRLLWPILG." Ion names and measured masses are indicated in the figure and are representative for $N = 2$ experiments.

© 2022 The Authors

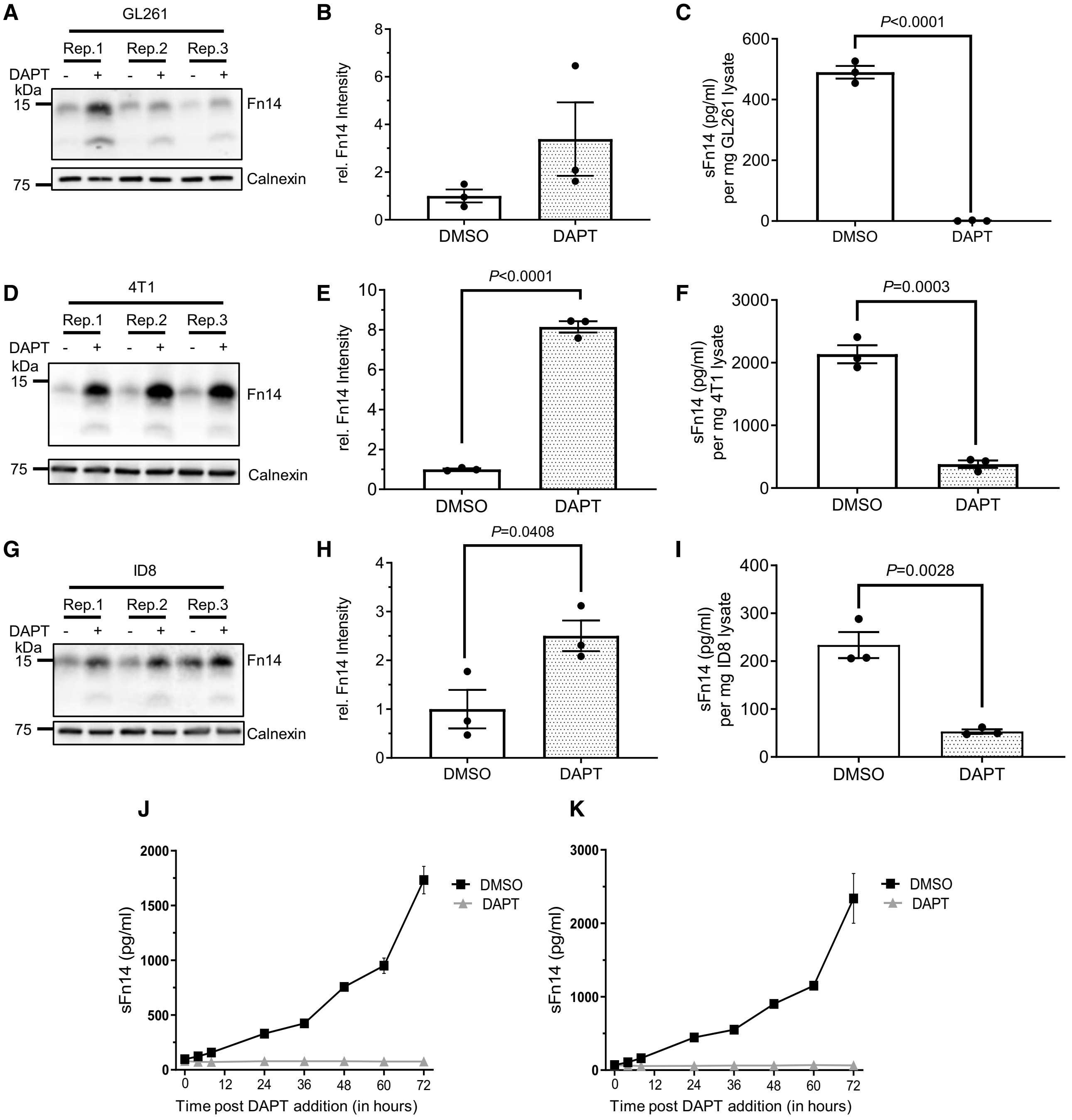
A
GL261
Rep.1 Rep.2 Rep.3
DAPT - + - + - +
kDa
15
Fn14
75
Calnexin
B
rel. Fn14 Intensity
DMSO DAPT
C
P<0.0001
sFn14 (pg/ml) per mg GL261 lysate
DMSO DAPT
D
4T1
Rep.1 Rep.2 Rep.3
DAPT - + - + - +
kDa
15
Fn14
75
Calnexin
E
P<0.0001
rel. Fn14 Intensity
DMSO DAPT
F
P=0.0003
sFn14 (pg/ml) per mg 4T1 lysate
DMSO DAPT
G
ID8
Rep.1 Rep.2 Rep.3
DAPT - + - + - +
kDa
15
Fn14
75
Calnexin
H
P=0.0408
rel. Fn14 Intensity
DMSO DAPT
I
P=0.0028
sFn14 (pg/ml) per mg ID8 lysate
DMSO DAPT
J
sFn14 (pg/ml)
Time post DAPT addition (in hours)
DMSO
DAPT
K
sFn14 (pg/ml)
Time post DAPT addition (in hours)
DMSO
DAPT

Figure EV2.

Figure EV2. Endogenous Fn14 processing in mouse cell lines by γ-secretase.

A Mouse glioblastoma cell line GL261 showed cellular accumulation of Fn14 upon γ-secretase inhibition. Cells were treated overnight with γ-secretase inhibitor DAPT (1 μM) or vehicle. Lysates of biological replicates (Rep.) were blotted for Fn14 with an antibody that targets the C-terminal end of the protein, or against calnexin as loading control.

B Quantification of blot in panel (A). Intensity values of Fn14 were normalized to the respective Calnexin loading control. The average of the control condition, where the cells were only treated with vehicle (DMSO), was consecutively normalized to 1.

C Conditioned media of the GL261 cells from panel (A) were collected, and sFn14 levels were measured by ELISA.

D Mouse breast cancer cell line 4T1 showed cellular accumulation of Fn14 upon γ-secretase inhibition. Cells were treated overnight with γ-secretase inhibitor DAPT (1 μM) or vehicle. Lysates were blotted for Fn14 with an antibody that targets the C-terminal end of the protein, or against calnexin as loading control.

E Quantification of blot in panel (D). Intensity values of Fn14 were normalized to the respective calnexin loading control. The average of the control condition, where the cells were only treated with vehicle (DMSO), was consecutively normalized to 1.

F Conditioned media of the 4T1 cells from panel (D) were collected and sFn14 levels measured by ELISA.

G Mouse ovarian cancer cell line ID8 showed cellular accumulation of Fn14 upon γ-secretase inhibition. Cells were treated overnight with γ-secretase inhibitor DAPT (1 μM) or vehicle. Lysates were blotted for Fn14 with an antibody that targets the C-terminal end of the protein, or against calnexin as loading control.

H Quantification of blot in panel (G). Intensity values of Fn14 were normalized to the respective calnexin loading control. The average of the control condition, where the cells were only treated with vehicle (DMSO), was consecutively normalized to 1.

I Conditioned media of the ID8 cells from panel (G) were collected and sFn14 levels measured by ELISA.

J, K Conditioned media of (J) MDA-MB-231 or (K) SKOV-3 cells were collected at indicated time points after DAPT (1 μM) or vehicle treatment and endogenous sFn14 levels were measured by ELISA. Even after 72 h DAPT still completely blocked γ-secretase, as evidenced by the lack of sFn14 secretion.

Data information: All quantification data are shown as mean ± SEM. The *P*-values that are above 0.05 have not been included into the panels. For all the panels, three biological replicates are performed. For panels (B), (C), (E), (F), (H), and (I), two-tailed unpaired *t*-tests were used. For panel (J) and (K), no statistical analysis was performed.

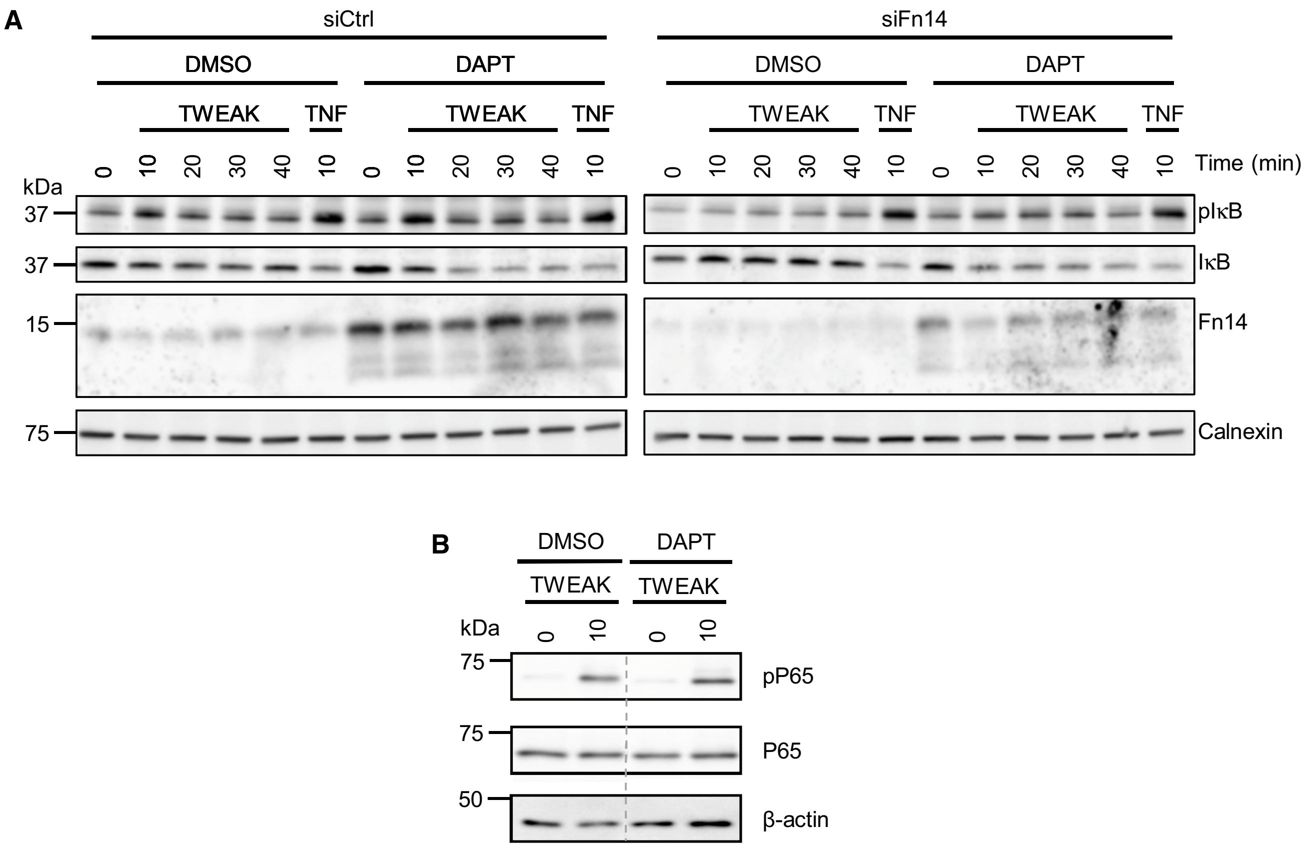

Figure EV3.

 © 2022 The Authors

Figure EV3. Inhibiting γ-secretase in MDA-MB-231 cells enhances Fn14 mediated NFκB signaling.

A MDA-MB-231 cells were transfected with an siRNA pool against human Fn14 or nontargeting control (Ctrl) siRNA. A day after transfection, cells were treated with γ-secretase inhibitor DAPT (1 μM) or vehicle overnight. Either TWEAK (100 ng/ml) or positive control TNF (10 ng/ml) were applied for indicated time points. The cell lysate was blotted against pIκB and IκB to evaluate NFκB activation or against Fn14 to verify the effect of the DAPT and siFn14 treatment, or against calnexin as a loading control. Shown are representative blots from $N = 4$ experiments.

B MDA-MB-231 cells were treated with γ-secretase inhibitor DAPT (1 μM) or vehicle overnight. TWEAK (100 ng/ml) was applied for 10 min. The cell lysate was blotted against pP65 and P65 to evaluate NFκB activation or against β-actin as a loading control. Shown are representative blots from $N = 3$ experiments. The dashed vertical line indicates that sample were run on the same blot but not directly next to each other.

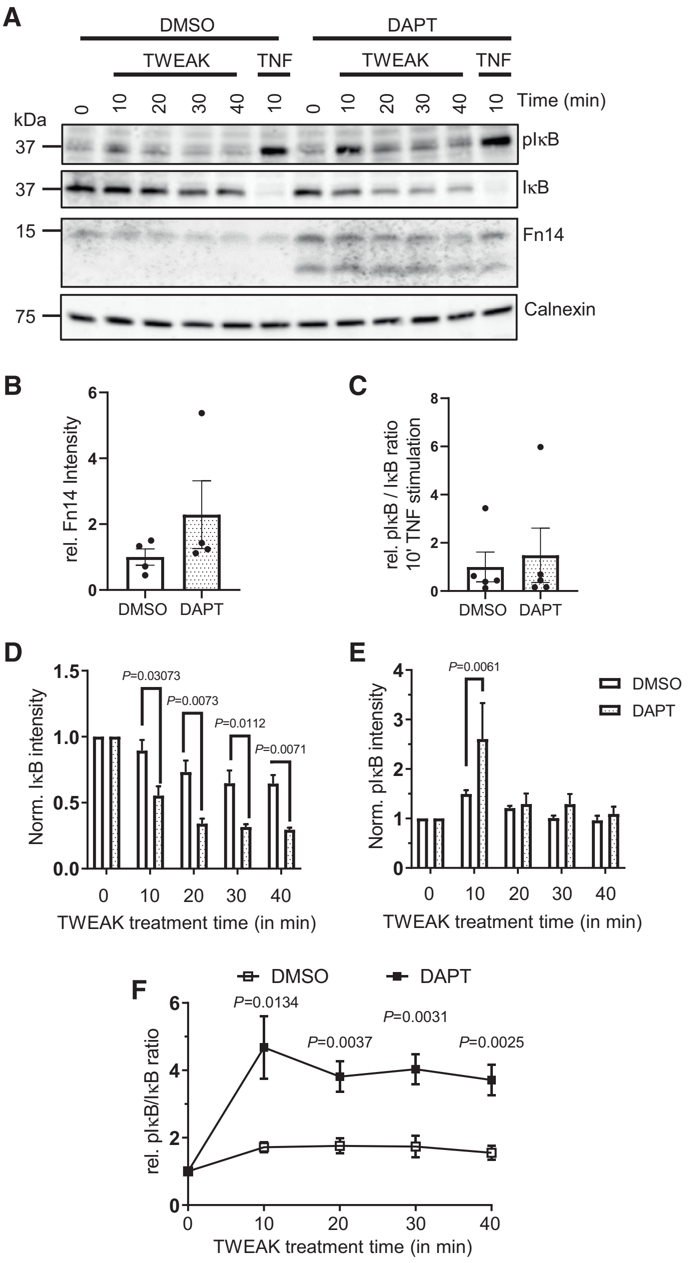

Figure EV4. Inhibiting γ-secretase in glioblastoma cell line U87 enhances Fn14 mediated NFκB signaling.

A U87 cells were treated with γ-secretase inhibitor DAPT (1 μM) or vehicle overnight. Either TWEAK (100 ng/ml) or positive control TNF (10 ng/ml) were applied for indicated time points. The cell lysate was blotted against pIκB and IκB to evaluate NFκB activation or against Fn14 to verify the effect of the DAPT and siFn14 treatment, or against calnexin as a loading control. Shown are representative blots from $N = 4–5$ experiments.

B U87 cells showed cellular accumulation of Fn14 upon γ-secretase inhibition. 0 min time point samples of Fn14 blot in panel (A) were quantified, normalized to the respective calnexin loading control and consecutively normalized to vehicle control average. Shown is the Fn14 intensity relative (rel.) to the DMSO control ($N = 4$).

C γ-Secretase inhibition by DAPT does not alter NFκB stimulation through TNF. U87 cells treated with TNF (10 ng/ml) for 10 min and the NFκB activation reported as ratio of pIκB to total IκB. Shown is the pIκB/IκB ratio relative (rel.) to the DMSO control ($N = 5$).

D, E Quantification of the IκB (D) and pIκB (E) blots in panel (A). The measurements were normalized to the 0 min time point. $N = 5$ biological replicates.

F The TWEAK stimulation of Fn14 and activation of NFκB is represented as ratio of pIκB to total IκB, taken from quantifications in (D) and (E). Shown is the pIκB/IκB ratio relative (rel.) to the 0 min time point. $N = 5$ biological replicates.

Data information: All quantification data are shown as mean ± SEM. The *P*-values that are above 0.05 have not been included into the panels. For panel (B) and (C), two-tailed unpaired *t*-tests were used. For panels (D) and (E), RM two-way ANOVAs with Šídák's multiple comparison test have been applied. For panel (F), two-tailed unpaired *t*-test have been applied for each time point. For all the panels, the number of biological replicates is reported in the corresponding panel legend.
