# Supplementary material for: Proteolytically generated soluble Tweak Receptor Fn14 is a blood biomarker for γ‐secretase activity
Source: EMBO Mol Med. 2022 Sep 7;14(10):e16084. doi: 10.15252/emmm.202216084 (PMC9549706; doi:10.15252/emmm.202216084)
Supplement: Supplementary file 5 [file EMMM-14-e16084-s001.pdf]

EMBO Molecular Medicine

Proteolytically generated soluble Tweak Receptor Fn14 is a blood biomarker for g-secretase activity

Gökhan Güner, Marlene Aßfalg, Kai Zhao, Tobias Dreyer, Shibojyoti Lahiri, Yun Lo, Biana Slivinschi, Axel Imhof, Georg Jocher, Laura Strohm, Christian Behrends, Dieter Langosch, Holger Bronger, Christopher Nimsky, Joerg Bartsch, Stanley Riddell, Harald Steiner, and Stefan Lichtenthaler
DOI: 10.15252/emmm.202216084

Corresponding author: Stefan Lichtenthaler (stefan.lichtenthaler@dzne.de)

Review Timeline:

Submission Date:	30th Mar 22
Editorial Decision:	10th May 22
Revision Received:	13th Jul 22
Editorial Decision:	22nd Jul 22
Revision Received:	29th Jul 22
Accepted:	15th Aug 22

Editor: Zeljko Durdevic

Transaction Report:

(Note: With the exception of the correction of typographical or spelling errors that could be a source of ambiguity, letters and reports are not edited. Depending on transfer agreements, referee reports obtained elsewhere may or may not be included in this compilation. Referee reports are anonymous unless the Referee chooses to sign their reports.)

10th May 2022

Dear Prof. Lichtenthaler,

Thank you for the submission of your manuscript to EMBO Molecular Medicine. We have now received feedback from the three reviewers who agreed to evaluate your manuscript. As you will see from the reports below, the referees acknowledge the interest of the study but also raise important concerns that should be addressed in a major revision.

Further consideration of a revision that addresses reviewers' concerns in full will entail a second round of review. EMBO Molecular Medicine encourages a single round of revision only and therefore, acceptance or rejection of the manuscript will depend on the completeness of your responses included in the next, final version of the manuscript. For this reason, and to save you from any frustrations in the end, I would strongly advise against returning an incomplete revision.

We would welcome the submission of a revised version within three months for further consideration. Please let us know if you require longer to complete the revision.

Please use this link to login to the manuscript system and submit your revision: Link Not Available

I look forward to receiving your revised manuscript.

Yours sincerely,

Zeljko Durdevic

Zeljko Durdevic
Editor
EMBO Molecular Medicine

***** Reviewer's comments *****

Referee #1 (Remarks for Author):

In this paper the authors provide evidence of the y-secretase processing of a cell surface receptor, Fn14, showing that Fn14 is another non-canonical y-secretase substrate that does not require ectodomain shedding, and reporting a novel role of y-secretase in regulating TWEAK/Fn14 signaling via NFKB pathway. The authors demonstrate that secreted Fn14 is easily measurable in plasma of both mice and humans and responds well to y-secretase inhibition. They propose that sFN14 can be used as a pharmacodynamic biomarker to monitor y-secretase activity ex vivo and in vivo. The paper is very well written, the findings are interesting, and the experiments are well designed and executed. One of the strengths of this paper is that the authors use and thoroughly characterize multiple models from cell lines, including patient derived cells, to plasma samples from mice and human patients, signifying physiological relevance of the findings.

Whereas the technical and biological aspects of the study are straightforward, and solid findings are supported by experimental data, the conclusion of therapeutic significance- that plasma sFn14 can be used as a pharmacodynamic in vivo biomarker "including in human tumor patients" - may not be fully substantiated. The sFn14 does not seem to be important diagnostic/predictability marker as there is no statistically significant difference in plasma sFn14 between the control and GBM patients (in neither initial nor recurrent diagnosis groups), or in patients with low vs. high ovarian cancer tumor burden. Although there was a difference in the serum sFn14 after treatment with GSI (controversial drug due to high toxicity/side-effects), the enthusiasm about therapeutic potential of the findings is somewhat dampened by limited applicability.

There are several mostly minor comments that should, however, be addressed. Specifically:

Fig. 1E In vitro y-secretase activity assay and 1G HEK 293 PSdko: Can authors comment why full-length Fn14 does not seem to be affected by 37C/4hrs or DAPT (in E) or by PS DKO (in G)? Especially, since later (Figs.3-6) they show, as one would expect, significantly increased amount of the FL Fn14 after inhibition of y-secretase by DAPT. Also, why did they choose to show C83 but not C99 in G? was C99 also accumulated in PS1 KO or PS DKO?

Fig. 3 shows that DAPT treatment increased the abundance of endogenous FL Fn14. However, in the text (page 5 last lane) the authors state: "suggesting...lack of full-length Fn14 upon DAPT treatment". Please clarify.

Table 1 is referred to in Methods but is not included in the manuscript files (at least in the files that I can see)
Fig. 8 is shown in the manuscript but is not referred to in the text.

DAPT treatment protocol: Could you clarify why in some experiments the authors use 24-48 hrs treatment (e.g. SKOV-3 cells) since DAPT stability is much shorter, just a few hours? Would a short-lived protein such as Fn14 recover and DAPT gets metabolized over 24-48hrs, thus the results of the 48hr DAPT treatment report something else? They have shown (Fig. 7A) that "The reduction [sFn14] was maintained at three and six hours after dosing and increased to the levels of the control-treated mice at the 24 h time point, consistent with the half-life of DAPT (Dovey et al., 2001)." Please clarify

Referee #2 (Remarks for Author):

TWEAK, a multi-functional member of the TNF family of cytokines, acts via binding to a cell surface receptor named Fn14. The TWEAK/Fn14 axis has been implicated in several physiological processes (e.g., wound repair) and in several diseases, including cancer. Here, Guner et al. report that Fn14 undergoes gamma-secretase mediated proteolytic cleavage that releases the extracellular domain into cell culture media (in vitro experiments) and into mouse/human plasma. They show that gamma-

secretase inhibition in vitro increases intracellular (Western blotting) and cell surface (flow cytometry) Fn14 levels, and they propose that this increase enhances TWEAK-triggered NF-kB activation. There is some very nice data in the manuscript, but there are also some issues that should be addressed. My comments on this manuscript are listed below:

Major comments:

1. Figures 3, 4, 5, 6, EV2, EV3- These figures all contain Western blot data examining endogenous Fn14 expression in cell lines/primary cells using a CST antibody generated against the Fn14 cytoplasmic tail. In Figures 3A and 3B, they put an asterisk next to a lower MW band and speculate as to what that immunoreactive protein might be. They say it might be an "alternative splice product induced by the lack of full length Fn14 upon DAPT treatment". Or it could be a "protease generated truncated Fn14 isoform". The fact is that there are many papers using many cell lines showing two main Fn14 bands in Western blots, with the smaller band most apparent in long film exposure times. You can see two or more bands in all of the Westerns shown here. It is unclear what these lower bands represent but there is a paper showing that MB-231 cells, used in some of the experiments here, express a splice variant mRNA that could produce a smaller Fn14 protein (Brown et al. 2013). This publication was not mentioned in the manuscript.

2. Figure 4- Why do Fn14 siRNA treatment experiments to "test the specificity of the Fn14 antibody? Is there a question about the quality of the CST antibody (Westerns) or the ITEM4 monoclonal (FACS)? These antibodies have been validated by the companies and used by many researchers.

3. Figure 5- Again why do Fn14 siRNA treatment experiments? The authors state that they need to "test if TWEAK is acting via Fn14". Does TWEAK cause downstream signaling by binding to some other protein? I would say the consensus in the field is that the only bona fide receptor is Fn14.

4. Figures 5, EV3- The authors propose that the data in these two figures supports the notion that gamma-secretase mediated proteolytic cleavage can regulate Fn14 signal transduction, and this is emphasized by including a schematic drawing for the final manuscript figure (Figure 8). This reviewer is not convinced by the results shown here. The authors only assay for one direct read-out of NF-kB pathway activation- phosphorylation of IkBa. There is a high basal level of p-IkBa in the cell lines (zero time point), the change in band intensity after TWEAK addition is small, and the difference between +/- DAPT treatment is also small. They do show an experiment looking at a downstream molecule - TNF- but still more experiments would need to be performed before one can conclude what is shown in Figure 8.

5. Figure 7- Panel A (mouse plasma samples) is a nice result but for the rest of the data it seems like the authors looked around for some available plasma/serum samples and did some Fn14 ELISAs. There is no statistically significant differences between the groups in Panel C (glioblastoma) and D (ovarian cancer) and I am not sure what to make of the multiple myeloma trial data with all the complexities of that type of drug study in humans. In any case, it is important to realize that the Fn14 detected in these ELISA assays may not necessarily be the gamma-secretase produced extracellular domain of the protein. In conclusion, at this time it may be a stretch to use the manuscript title "Proteolytically generated soluble Tweak receptor Fn14 is a blood biomarker for gamma-secretase activity".

Minor comments:

1. Figure 1- The authors should consider moving the panel D drawing to the end of the figure so they can refer to it after presenting all the supporting data in the other panels.
2. On page 4, the authors state that "although ADAM proteases shed several TNFRs" but they do not include any references.

3. Throughout the paper the authors refer to Tran et al. 2006 when they mention that Fn14 is overexpressed in GBM. There have been numerous more recent publications confirming and extending this 16-year-old data.

4. The Discussion is very, very long.

Referee #3 (Comments on Novelty/Model System for Author):

Identification of Fn14 as a biomarker for y-secretase is clinically important as this protease plays major role in various diseases. To identify whether inhibitors to y-secretase is working would be clinically significant. Model systems are appropriate but with more clinical validation (beyond the scope of this manuscript) would strengthen the study.

Referee #3 (Remarks for Author):

Guner et al.

The manuscript by Guner et al. showed that the Fn14 receptor is a target for y-secretase. Biochemical studies revealed that y-secretase cleaved Fn14 within the transmembrane domain. Inhibition of y-secretase resulted in an enhance expression of Fn14 at the surface and activation of Fn14 by TWEAK. Ex vivo analysis showed that secreted Fn14 is highly detected in the serum of GBM patients. Moreover, secreted Fn14 is reduced in patients dosed with y-secretase inhibitors prior to CAR(T) therapy. Overall, the mechanistic studies are straightforward, well designed and written with the novelty finding of Fn14 secretion as a potential readout for y-secretase. Some minor concerns are noted:

1. If the y-secretase region is known or peptide sequence is identified, the mutation studies to the y-secretase region of Fn14 would enhance the concept that this is solely mediated by y-secretase.
2. The discussion section, it is unclear how measurable sFn14 would help patients with AD, considering Fn14 expression is not highly detected in AD. The authors should revised the claim or expand on the concept.
3. Discussion section is long could be reduced.

We thank all three Referees for their encouraging and constructive reviews. A point-by-point response to each of their comments is provided below. At the end of the document we also add our answers and comments to the formatting points raised by the editorial office.

***** Reviewer's comments *****

Referee #1 (Remarks for Author):

In this paper the authors provide evidence of the γ-secretase processing of a cell surface receptor, Fn14, showing that Fn14 is another non-canonical γ-secretase substrate that does not require ectodomain shedding, and reporting a novel role of γ-secretase in regulating TWEAK/Fn14 signaling via NFKB pathway. The authors demonstrate that secreted Fn14 is easily measurable in plasma of both mice and humans and responds well to γ-secretase inhibition. They propose that sFN14 can be used as a pharmacodynamic biomarker to monitor γ-secretase activity ex vivo and in vivo. The paper is very well written, the findings are interesting, and the experiments are well designed and executed. One of the strengths of this paper is that the authors use and thoroughly characterize multiple models from cell lines, including patient derived cells, to plasma samples from mice and human patients, signifying physiological relevance of the findings.

Whereas the technical and biological aspects of the study are straightforward, and solid findings are supported by experimental data, the conclusion of therapeutic significance- that plasma sFn14 can be used as a pharmacodynamic in vivo biomarker "including in human tumor patients" - may not be fully substantiated.
The sFn14 does not seem to be important diagnostic/predictability marker as there is no statistically significant difference in plasma sFn14 between the control and GBM patients (in neither initial nor recurrent diagnosis groups), or in patients with low vs. high ovarian cancer tumor burden. Although there was a difference in the serum sFn14 after treatment with GSI (controversial drug due to high toxicity/side-effects), the enthusiasm about therapeutic potential of the findings is somewhat dampened by limited applicability.
We agree with this reviewer: sFn14 does not appear to be a diagnostic marker, at least not for the two tumors tested in our study. However, we demonstrate that sFn14 is suitable as a blood-based therapy-response marker for gamma-secretase inhibitors. Such a so-called pharmacodynamic activity marker is urgently needed. And this is what we claim in the title and the abstract.

There are several mostly minor comments that should, however, be addressed. Specifically:

Fig. 1E In vitro γ-secretase activity assay and 1G HEK 293 PSdko: Can authors comment why full-length Fn14 does not seem to be affected by 37C/4hrs or DAPT (in E) or by PS DKO (in G)? Especially, since later (Figs.3-6) they show, as one would expect, significantly increased amount of the FL Fn14 after inhibition of γ-secretase by DAPT. Also, why did they choose to show C83 but not C99 in G? was C99 also accumulated in PS1 KO or PS DKO?
Our transient overexpression of Fn14 in HEK cells (Fig. 1) was so efficient, that the transfected protein abundance was many folds higher compared to endogenous Fn14 protein analyzed in Figs. 3-6. Because we saw the DAPT-dependent enrichment of full-length

Fn14 only for endogenous, but not for strongly overexpressed Fn14, we conclude that the overexpressed Fn14 levels are so high that only a fraction of that protein is available for gamma-secretase cleavage, so that inhibition of gamma-secretase with DAPT does not lead to a significant increase in full-length Fn14, while the levels of the gamma-secretase-cleaved sFn14 are obviously strongly reduced. We see this also for proteins other than Fn14 when we strongly overexpress them.

C99 also accumulated in the PS DKO cells, but had a lower abundance compared to C83, in agreement with the initial DAPT publication (Dovey et al. J Neurochem 2001). We have now added to Fig. 1F a longer exposure of our blot and show C99 and C83 in the same blot. It is known that upon gamma-secretase inhibition, C99 can be increasingly cleaved by alpha-secretase, which converts C99 to C83 (e.g. Kuhn et al. EMBO J 2010). Additionally, APP in HEK293 cells is cleaved to a larger extent by alpha- compared to beta-secretase, resulting in higher C83 than C99 levels.

Fig. 3 shows that DAPT treatment increased the abundance of endogenous FL Fn14. However, in the text (page 5 last lane) the authors state: "suggesting...lack of full-length Fn14 upon DAPT treatment". Please clarify.
We have corrected this sentence. The two words “cleavage of” were missing. The corrected sentence on top of page 6 is: “suggesting that it is either an alternative splice form of Fn14 (Brown *et al*, 2013) induced by the lack of cleavage of full-length Fn14 upon DAPT treatment or that it is truncated by a protease that is not inhibited by TAPI-1.”

Table 1 is referred to in Methods but is not included in the manuscript files (at least in the files that I can see)
We corrected this mistake. The data is not shown in a separate table, but instead in Fig. 2B. This is now correctly mentioned in the methods section on MALDI on top of page 11.

Fig. 8 is shown in the manuscript but is not referred to in the text.
We corrected this mistake. The figure is now referred to in the first paragraph of the discussion on page 8.

DAPT treatment protocol: Could you clarify why in some experiments the authors use 24-48 hrs treatment (e.g. SKOV-3 cells) since DAPT stability is much shorter, just a few hours? Would a short-lived protein such as Fn14 recover and DAPT gets metabolized over 24-48hrs, thus the results of the 48hr DAPT treatment report something else? They have shown (Fig. 7A) that "The reduction [sFn14] was maintained at three and six hours after dosing and increased to the levels of the control-treated mice at the 24 h time point, consistent with the half-life of DAPT (Dovey et al., 2001)." Please clarify
The half-life of DAPT is up to 24 h in vivo and not longer, because of DAPT metabolism and excretion in vivo. We have now added on page 7 the two words “in vivo” to the sentence mentioned by the reviewer: “The reduction [sFn14] was maintained at three and six hours after dosing and increased to the levels of the control-treated mice at the 24 h time point, consistent with the in vivo half-life of DAPT (Dovey et al., 2001)."
In contrast, in cultured cells the half-life of DAPT is not known and there is no reason to assume that DAPT would not be active anymore after one day. To address this issue we have conducted an experiment where we treated SKOV-3 and MDA-MB-231 for 72 h with DAPT and collected aliquots of the same medium at different time points. While sFn14

concentration increased over time in DMSO-treated control cells, there was no increase seen in the presence of DAPT, indicating efficient inhibition of gamma-secretase for the whole time course. This data is now added as Fig. EV2J, K. The corresponding sentence in the results section at the bottom of page 5 is "DAPT blocked sFn14 efficiently over a time course of 72 h, as determined in MDA-MB-231 and SKOV-3 cells (Fig. EV2J, K)."

Referee #2 (Remarks for Author):

TWEAK, a multi-functional member of the TNF family of cytokines, acts via binding to a cell surface receptor named Fn14. The TWEAK/Fn14 axis has been implicated in several physiological processes (e.g., wound repair) and in several diseases, including cancer. Here, Guner et al. report that Fn14 undergoes gamma-secretase mediated proteolytic cleavage that releases the extracellular domain into cell culture media (in vitro experiments) and into mouse/human plasma. They show that gamma-secretase inhibition in vitro increases intracellular (Western blotting) and cell surface (flow cytometry) Fn14 levels, and they propose that this increase enhances TWEAK-triggered NF-kB activation. There is some very nice data in the manuscript, but there are also some issues that should be addressed. My comments on this manuscript are listed below:

Major comments:

1. Figures 3, 4, 5, 6, EV2, EV3- These figures all contain Western blot data examining endogenous Fn14 expression in cell lines/primary cells using a CST antibody generated against the Fn14 cytoplasmic tail. In Figures 3A and 3B, they put an asterisk next to a lower MW band and speculate as to what that immunoreactive protein might be. They say it might be an "alternative splice product induced by the lack of full length Fn14 upon DAPT treatment". Or it could be a "protease generated truncated Fn14 isoform". The fact is that there are many papers using many cell lines showing two main Fn14 bands in Western blots, with the smaller band most apparent in long film exposure times. You can see two or more bands in all of the Westerns shown here. It is unclear what these lower bands represent but there is a paper showing that MB-231 cells, used in some of the experiments here, express a splice variant mRNA that could produce a smaller Fn14 protein (Brown et al. 2013). This publication was not mentioned in the manuscript.
This reference is now included. The new sentence at the top of page 6 is now: "suggesting that it is either an alternative splice form of Fn14 (Brown *et al*, 2013) induced by the lack of cleavage of full-length Fn14 upon DAPT treatment or that it is truncated by a protease that is not inhibited by TAPI-1."

2. Figure 4- Why do Fn14 siRNA treatment experiments to "test the specificity of the Fn14 antibody? Is there a question about the quality of the CST antibody (Westerns) or the ITEM4 monoclonal (FACS)? These antibodies have been validated by the companies and used by many researchers.
We are aware that these antibodies are widely used and specific. Because we used the antibodies for the first time in our group, we took extra care to ensure that we used the antibodies correctly. For that reason, we included the siRNA experiments. In the manuscript, we have now modified the corresponding sentence on page 6. Instead of writing "As a control for the specificity of the antibody,", we now simply write "As expected,…".

3. Figure 5- Again why do Fn14 siRNA treatment experiments? The authors state that they need to "test if TWEAK is acting via Fn14". Does TWEAK cause downstream signaling by binding to some other protein? I would say the consensus in the field is that the only bona fide receptor is Fn14.
Similar to point 2 above, we modified the corresponding sentence on page 6. We deleted the last part of the sentence (“demonstrating that the TWEAK-induced NFkB activation was mediated through Fn14”) and added the words “as expected” to the beginning of that same sentence (As expected, siRNA-mediated knock-down of Fn14 reduced…).

4. Figures 5, EV3- The authors propose that the data in these two figures supports the notion that gamma-secretase mediated proteolytic cleavage can regulate Fn14 signal transduction, and this is emphasized by including a schematic drawing for the final manuscript figure (Figure 8). This reviewer is not convinced by the results shown here. The authors only assay for one direct read-out of NF-kB pathway activation- phosphorylation of IkBa. There is a high basal level of p-IkBa in the cell lines (zero time point), the change in band intensity after TWEAK addition is small, and the difference between +/- DAPT treatment is also small. They do show an experiment looking at a downstream molecule - TNF- but still more experiments would need to be performed before one can conclude what is shown in Figure 8.
We appreciate this reviewer’s comment and added more experiments, as pointed out in the following. Additionally, we would like to emphasize that gamma-secretase cleavage is not an essential step for Fn14 signaling or for completely suppressing Fn14 signaling. In contrast to essential signaling components, such as Tweak or Fn14 itself, we view gamma-secretase as having a modulatory function on Fn14 abundance and consequently on Fn14 signaling. It is clear that we can block or delete gamma-secretase – but that Fn14 signaling still occurs, but to a slightly enhanced level compared to the control condition. In line with this careful data interpretation we toned down the relevant sections in the manuscript, where we now write that “gamma-secretase affects Fn14 signaling” (bottom of page 6) instead of “gamma-secretase controls Fn14 signaling”. In the first paragraph of the discussion we describe gamma-secretase as a “new modulator” and not “new regulator” of Fn14 signaling.
In addition to the IkB/pIkB measurements and TNF secretion as a result of Fn14 signaling we now demonstrate that TWEAK stimulates NFkB P65 phosphorylation and downstream signal transduction, using an NFKB-dependent luciferase reporter assay. Importantly, in both assays gamma-secretase inhibition increased the signal by up to two-fold – consistent with the experiments already provided in the previous version of the manuscript. The new panel on NFkB phosphorylation is included as new Fig. EV3 (for the Western blots) and with the quantification in main Fig. 5, panel E. To save space in the main figure, the IkB Western blots were also moved to Fig. EV3. The luciferase reporter assay is now described in Fig. 5G and in the results on page 6:”As an additional read-out for downstream signaling we transfected SKOV-3 cells with an established reporter construct expression luciferase under the control of a NFkB-responsive promoter. Addition of TWEAK stimulated luciferase expression and this increase was enhanced by 60% upon DAPT treatment (Fig. 5G)”.

5. Figure 7- Panel A (mouse plasma samples) is a nice result but for the rest of the data it seems like the authors looked around for some available plasma/serum samples and did some Fn14 ELISAs. There is no statistically significant differences between the groups in

Panel C (glioblastoma) and D (ovarian cancer) and I am not sure what to make of the multiple myeloma trial data with all the complexities of that type of drug study in humans. In any case, it is important to realize that the Fn14 detected in these ELISA assays may not necessarily be the gamma-secretase produced extracellular domain of the protein. In conclusion, at this time it may be a stretch to use the manuscript title "Proteolytically generated soluble Tweak receptor Fn14 is a blood biomarker for gamma-secretase activity".
We agree with this reviewer: sFn14 does not appear to be a diagnostic marker, at least not for the two tumors tested in our study that are characterized by high Fn14 expression. However, we demonstrate that sFn14 is suitable as a blood-based therapy-response marker for gamma-secretase inhibitors, as shown in mice and human. Such a marker is referred to as a pharmacodynamic activity marker for gamma-secretase and this is urgently needed. This is also what we claim in the title and the abstract, where we talk about an activity marker and not a diagnostic marker.
Regarding the length of the sFn14 protein: We find that sFn14 is generated in a gamma-secretase-dependent manner in human and mouse cell lines, in primary human cells and in mice and in humans. Moreover, we demonstrate that sFn14 secreted from a human cell line corresponds exactly to the expected gamma-secretase cleavage product, as seen by its molecular weight on an immunoblot and by mass spectrometry-based sequencing. Additionally, Fn14 fulfills all general criteria required for a gamma-secretase substrate, such as being a type I membrane protein. Thus, the most simple explanation for the reduction of sFn14 in blood after gamma-secretase inhibition is that sFn14 is indeed the gamma-secretase-truncated sFn14 form comprising its ectodomain plus a part of its transmembrane domain. Yet, following the reviewer’s comment we also tried to address the length of plasma sFn14 with direct experiments. We immunoprecipitated sFn14 from human plasma using an N-terminal antibody. We do indeed detect a protein band at a molecular weight between 5 and 10 kDa on a Coomassie gel (see picture below), in line with a gamma-secretase cleavage within the Fn14 ectodomain. We cut out this band from the Schägger gel and subjected it after proteolytic digest to mass spectrometric analysis. We identified one Fn14 peptide. Importantly, this peptide is derived from the Fn14 ectodomain, as expected for a gamma-secretase-cleaved sFn14 in plasma and in line with the molecular weight below 10 kDa. Yet, the recovery of the protein was obviously not very efficient, because we only detected a single peptide from Fn14. To firmly conclude that this band does not give rise to cytoplasmic Fn14 peptides and therefore must be sFn14, we first need to improve our IP conditions for Fn14 in order to be able to detect more Fn14-derived peptides, ideally covering the whole ectodomain. We realize that this will take substantially more time and thus, decided to show this preliminary data only here in the rebuttal letter and not in the manuscript.

Figure for reviewers removed

Minor comments:

1. Figure 1- The authors should consider moving the panel D drawing to the end of the figure so they can refer to it after presenting all the supporting data in the other panels.
We appreciate this suggestion, but prefer to leave the panel as panel D instead of as panel G, because many readers of the manuscript will not be experts in gamma-secretase. Thus, we feel that illustrating what the ICD is (relevant for panel E) and what the catalytic subunit of gamma-secretase is (relevant for panels F and G) may help the readers to follow the experiments more easily.

2. On page 4, the authors state that "although ADAM proteases shed several TNFRs" but they do not include any references.
As suggested, we included relevant references on page 4, e.g. for TNFR1 (Reddy JBC 2000), TNFR2/p75 (Peschon Science 1998), p75 NTR (Weskamp JBC04) and DR6 (Colombo EMBO 2018)

3. Throughout the paper the authors refer to Tran et al. 2006 when they mention that Fn14 is overexpressed in GBM. There have been numerous more recent publications confirming and extending this 16-year-old data.
As suggested, we added on top of page 7 two newer citations: Hersh et al. Neuro Oncol 2018 and Perez et al. Oncogene, 2016.

4. The Discussion is very, very long.
As suggested, we shortened the discussion – from 3 pages to 2 pages.

Referee #3 (Remarks for Author):

The manuscript by Guner et al. showed that the Fn14 receptor is a target for γ-secretase. Biochemical studies revealed that γ-secretase cleaved Fn14 within the transmembrane domain. Inhibition of γ-secretase resulted in an enhance expression of Fn14 at the surface and activation of Fn14 by TWEAK. Ex vivo analysis showed that secreted Fn14 is highly detected in the serum of GBM patients. Moreover, secreted Fn14 is reduced in patients dosed with γ-secretase inhibitors prior to CAR(T) therapy. Overall, the mechanistic studies are straightforward, well designed and written with the novelty finding of Fn14 secretion as a potential readout for γ-secretase. Some minor concerns are noted:

1. If the γ-secretase region is known or peptide sequence is identified, the mutation studies to the γ-secretase region of Fn14 would enhance the concept that this is solely mediated by γ-secretase.

gamma-Secretase – in contrast to some other proteases, such as trypsin or caspases – does not have a specific cleavage site within the transmembrane domain of its substrates. This has been demonstrated in numerous publications using diverse gamma-secretase substrates, in particular APP and Notch. In fact, mutations within the transmembrane domain may affect the specific cleavage site, but rarely the total amount of cleavage. Thus, it is not possible to do rationale design and mutate single amino acids within the Fn14 transmembrane domain with the aim of blocking gamma-secretase cleavage.

2. The discussion section, it is unclear how measurable sFn14 would help patients with AD, considering Fn14 expression is not highly detected in AD. The authors should revised the claim or expand on the concept.

We fully agree with this reviewer. There is no evidence that sFn14 measurement would be useful for AD patients, for example as a diagnostic biomarker. The point that we want to make is the following. Intensive research is ongoing to try to develop gamma-secretase inhibitors (GSIs) that block gamma-secretase in a substrate-selective manner so that only (or preferentially) the cleavage of the AD-linked APP protein is blocked (and thus, Abeta generation), but not the cleavage of Notch or other substrates, such as Fn14. This may be an excellent way to prevent the side effects of long-term gamma-secretase inhibition. As mentioned in the manuscript, it is difficult to monitor gamma-secretase activity in vivo in blood. Thus, development of APP-selective (or -preferring) GSIs may benefit from measuring sFn14 in blood. If such a GSI blocks generation of Abeta and also of sFn14, it is obviously not substrate-specific. Thus, measurement of sFn14 may be used a surrogate marker for substrate-selective gamma-secretase inhibition. Because this reviewer (next point) and reviewer 2 suggest to shorten the discussion, we did not expand on the topic of substrate-selective gamma-secretase inhibition in the manuscript. The sentence in the discussion on page 9 is now: "Additionally, sFn14 may help to develop substrate-preferring GSIs which preferentially block generation of the Abeta peptide in AD over the cleavage of other substrates, such as Fn14, and, thus, avoid the side effects observed upon chronic GSI treatment.".

3. Discussion section is long could be reduced.

As suggested, we shortened the discussion – from 3 pages to 2 pages.

22nd Jul 2022

Dear Prof. Lichtenthaler,

Thank you for the submission of your revised manuscript to EMBO Molecular Medicine. I am pleased to inform you that we will be able to accept your manuscript pending the following final amendments:

1) Please address all the minor points raised by the referee.
2) In the main manuscript file, please do the following:
- Correct/answer the track changes suggested by our data editors by working from the attached document.
- Limit Keywords to max.5.
- In M&M, add statistical paragraph that should reflect all information that you have filled in the Authors Checklist, especially regarding randomization, blinding, replication.
- In M&M, please include statement that in addition to WMA Declaration of Helsinki the experiments also conformed the Department of Health and Human Services Belmont Report.
- Please rename "Conflict of Interest" to "Disclosure Statement & Competing Interests". We updated our journal's competing interests policy in January 2022 and request authors to consider both actual and perceived competing interests. Please review the policy https://www.embopress.org/competing-interests and update your competing interests if necessary.
3) Author contributions: Please specify author contributions in our submission system. CRediT has replaced the traditional author contributions section because it offers a systematic machine readable author contributions format that allows for more effective research assessment. You are encouraged to use the free text boxes beneath each contributing author's name to add specific details on the author's contribution. More information is available in our guide to authors: https://www.embopress.org/page/journal/17574684/authorguide#authorshipguidelines
4) Add "The Paper Explained" to the main manuscript file.
5) Synopsis: Please check your synopsis text and image before submission with your revised manuscript. Please be aware that in the proof stage minor corrections only are allowed (e.g., typos).
6) For more information: Please remove corresponding author's e-mail address. This space should be used to list relevant web links for further consultation by our readers. Could you identify some relevant ones and provide such information as well? Some examples are patient associations, relevant databases, OMIM/proteins/genes links, author's websites, etc...
7) Source data: We encourage you to include the source data for figure panels that show essential data. Numerical data should be provided as individual .xls or .csv files (including a tab describing the data). For blots or microscopy, uncropped images should be submitted (using a zip archive if multiple images need to be supplied for one panel). For the main figures please upload one file per figure, while all EV figures should be zipped and uploaded as one file. Please check "Author Guidelines" for more information. https://www.embopress.org/page/journal/17574684/authorguide#sourcedata
8) As part of the EMBO Publications transparent editorial process initiative (see our Editorial at http://embomolmed.embopress.org/content/2/9/329), EMBO Molecular Medicine will publish online a Review Process File (RPF) to accompany accepted manuscripts. This file will be published in conjunction with your paper and will include the anonymous referee reports, your point-by-point response and all pertinent correspondence relating to the manuscript. Let us know whether you agree with the publication of the RPF and as here, if you want to remove or not any figures from it prior to publication. Please note that the Authors checklist will be published at the end of the RPF.
9) Please provide a point-by-point letter INCLUDING my comments as well as the reviewer's reports and your detailed responses (as Word file).

I look forward to reading a new revised version of your manuscript as soon as possible.

Yours sincerely,

Zeljko Durdevic

***** Reviewer's comments *****

Referee #2 (Remarks for Author):

The authors responded well to my previous critique, and as such this is a much improved paper. I found three minor problems in the revised manuscript text.

1. Page 7, last sentence of the top paragraph: As mentioned in my previous review, that lower MW band may not be a cleavage product, but an alternative splicing isoform. Please edit the sentence.

2. Page 7, first sentence of third paragraph: As mentioned in my previous review, there are many newer references than Tran 2006, including a review in Oncogene, showing that Fn14 is overexpressed in GBM tumors. Please edit the sentence.

3. Page 8 last sentence that continues onto top of page 9: The authors state that "Surface Fn14 is used as a target for the delivery of immunotoxins..." and then they cite 5 publications. Two of those publications are not immunotoxin papers. Dancy et al describes Fn14-targeted, drug loaded nanoparticle delivery and Lerchen et al describes antibody-drug conjugates. Please edit the sentence.

Point-by-point response letter

Dear Dr. Durdevic,

Please find below our response in blue letters.

1) Please address all the minor points raised by the referee.
We have addressed all points as described at the end of this document.

2) In the main manuscript file, please do the following:
All our changes to your "data edited" manuscript file are shown in track changes.
- Correct/answer the track changes suggested by our data editors by working from the attached document.
Done.
- Limit Keywords to max.5.
Done.
- In M&M, add statistical paragraph that should reflect all information that you have filled in the Authors Checklist, especially regarding randomization, blinding, replication.
We have added a short paragraph at the very end of the M&M section on page 15 and hope to have considered all relevant points.
- In M&M, please include statement that in addition to WMA Declaration of Helsinki the experiments also conformed the Department of Health and Human Services Belmont Report.
Done.
- Please rename "Conflict of Interest" to "Disclosure Statement & Competing Interests". We updated our journal's competing interests policy in January 2022 and request authors to consider both actual and perceived competing interests. Please review the policy https://www.embopress.org/competing-interests and update your competing interests if necessary.
Done.

3) Author contributions: Please specify author contributions in our submission system. CRediT has replaced the traditional author contributions section because it offers a systematic machine readable author contributions format that allows for more effective research assessment. You are encouraged to use the free text boxes beneath each contributing author's name to add specific details on the author's contribution. More information is available in our guide to authors:https://www.embopress.org/page/journal/17574684/authorguide#authorshipguidelines
Done. This was already included last time. We hope that this is correct.

4) Add "The Paper Explained" to the main manuscript file.
Done.

5) Synopsis: Please check your synopsis text and image before submission with your revised manuscript. Please be aware that in the proof stage minor corrections only are allowed (e.g., typos).

******* Reviewer's comments *******

Referee #2 (Remarks for Author):

The authors responded well to my previous critique, and as such this is a much improved paper. I found three minor problems in the revised manuscript text.

1. Page 7, last sentence of the top paragraph: As mentioned in my previous review, that lower MW band may not be a cleavage product, but an alternative splicing isoform. Please edit the sentence.
We have added the comment as suggested on top of (new) page 8. The new sentence is now: “DAPT treatment also induced generation of the alternative, lower-molecular weight form of Fn14 (Fig. 6A), which may be an alternative splice form of Fn14 (Brown *et al*, 2013) or a proteolytically truncated form.”

2. Page 7, first sentence of third paragraph: As mentioned in my previous review, there are many newer references than Tran 2006, including a review in Oncogene, showing that Fn14 is overexpressed in GBM tumors. Please edit the sentence.
We have added additional papers as suggested in the middle of (new) page 8. The new sentence is now:”Next, we tested whether sFn14 is also detectable in human blood by ELISA and how it differs among healthy individuals and among individuals with GBM, where Fn14 shows increased expression (Hersh *et al*, 2018; Perez *et al.*, 2016; Tran *et al*, 2006).” This does include the suggested Oncogene review.

3. Page 8 last sentence that continues onto top of page 9: The authors state that "Surface Fn14 is used as a target for the delivery of immunotoxins..." and then they cite 5 publications. Two of those publications are not immunotoxin papers. Dancy et al describes Fn14-targeted, drug loaded nanoparticle delivery and Lerchen et al describes antibody-drug conjugates. Please edit the sentence.
We have edited the sentence as suggested. The new sentence at the bottom of (new) page 10 is now:“Surface Fn14 is used as a target for the delivery of immunotoxins and antibody-drug conjugates to reduce the number of Fn14-expressing tumor cells (Alvarez de Cienfuegos *et al.*, 2020; Dancy *et al.*, 2020; Lerchen *et al*, 2018; Zhou *et al*, 2013; Zhou *et al.*, 2014).”

We are pleased to inform you that your manuscript is accepted for publication and is now being sent to our publisher to be included in the next available issue of EMBO Molecular Medicine.

EMBO Press Author Checklist

Corresponding Author Name: Stefan F. Lichtenthaler
Journal Submitted to: EMBO Molecular Medicine
Manuscript Number: EMM-2022-16084

USEFUL LINKS FOR COMPLETING THIS FORM
The EMBO Journal - Author Guidelines
EMBO Reports - Author Guidelines
Molecular Systems Biology - Author Guidelines
EMBO Molecular Medicine - Author Guidelines

Reporting Checklist for Life Science Articles (updated January 2022)
This checklist is adapted from Materials Design Analysis Reporting (MDAR) Checklist for Authors. MDAR establishes a minimum set of requirements in transparent reporting in the life sciences (see Statement of Task: 10.31222/osf.io/9sm4x). Please follow the journal's guidelines in preparing your manuscript.
Please note that a copy of this checklist will be published alongside your article.

Abridged guidelines for figures
1. Data
The data shown in figures should satisfy the following conditions:
- the data were obtained and processed according to the field's best practice and are presented to reflect the results of the experiments in an accurate and unbiased manner.
- ideally, figure panels should include only measurements that are directly comparable to each other and obtained with the same assay.
- plots include clearly labeled error bars for independent experiments and sample sizes. Unless justified, error bars should not be shown for technical replicates.
- if n<5, the individual data points from each experiment should be plotted. Any statistical test employed should be justified.
- Source Data should be included to report the data underlying figures according to the guidelines set out in the authorship guidelines on Data Presentation.

2. Captions
Each figure caption should contain the following information, for each panel where they are relevant:
- a specification of the experimental system investigated (eg cell line, species name).
- the assay(s) and method(s) used to carry out the reported observations and measurements.
- an explicit mention of the biological and chemical entity(ies) that are being measured.
- an explicit mention of the biological and chemical entity(ies) that are altered/varied/perturbed in a controlled manner.
- the exact sample size (n) for each experimental group/condition, given as a number, not a range;
- a description of the sample collection allowing the reader to understand whether the samples represent technical or biological replicates (including how many animals, litters, cultures, etc.).
- a statement of how many times the experiment shown was independently replicated in the laboratory.
- definitions of statistical methods and measures:
  - common tests, such as t-test (please specify whether paired vs. unpaired), simple χ2 tests, Wilcoxon and Mann-Whitney tests, can be unambiguously identified by name only, but more complex techniques should be described in the methods section;
  - are tests one-sided or two-sided?
  - are there adjustments for multiple comparisons?
  - exact statistical test results, e.g., P values = x but not P values < x;
  - definition of 'center values' as median or average;
  - definition of error bars as s.d. or s.e.m.

Please complete ALL of the questions below.
Select "Not Applicable" only when the requested information is not relevant for your study.

Materials

Newly Created Materials	Information included in the manuscript?	In which section is the information available? (Reagents and Tools Table, Materials and Methods, Figures, Data Availability Section)
New materials and reagents need to be available; do any restrictions apply?	Not Applicable	

Antibodies	Information included in the manuscript?	In which section is the information available? (Reagents and Tools Table, Materials and Methods, Figures, Data Availability Section)
For **antibodies** provide the following information:<br>- Commercial antibodies: RRID (if possible) or supplier name, catalogue number and or/clone number<br>- Non-commercial: RRID or citation	Yes	Materials and Methods

DNA and RNA sequences	Information included in the manuscript?	In which section is the information available? (Reagents and Tools Table, Materials and Methods, Figures, Data Availability Section)
**Short novel DNA or RNA including primers, probes**: provide the sequences.	Not Applicable	

Cell materials	Information included in the manuscript?	In which section is the information available? (Reagents and Tools Table, Materials and Methods, Figures, Data Availability Section)
**Cell lines:** Provide species information, strain. Provide accession number in repository **OR** supplier name, catalog number, clone number, and/**OR** RRID.	Yes	Materials and Methods
**Primary cultures:** Provide species, strain, sex of origin, genetic modification status.	Yes	Materials and Methods
Report if the cell lines were recently **authenticated** (e.g., by STR profiling) and tested for mycoplasma contamination.	Yes	Materials and Methods

Experimental animals	Information included in the manuscript?	In which section is the information available? (Reagents and Tools Table, Materials and Methods, Figures, Data Availability Section)
**Laboratory animals or Model organisms:** Provide species, strain, sex, age, genetic modification status. Provide accession number in repository **OR** supplier name, catalog number, clone number, **OR** RRID.	Yes	Materials and Methods
**Animal observed in or captured from the field:** Provide species, sex, and age where possible.	Not Applicable	
Please detail **housing and husbandry conditions**.	Yes	Materials and Methods

Plants and microbes	Information included in the manuscript?	In which section is the information available? (Reagents and Tools Table, Materials and Methods, Figures, Data Availability Section)
**Plants:** provide species and strain, ecotype and cultivar where relevant, unique accession number if available, and source (including location for collected wild specimens).	Not Applicable	
**Microbes:** provide species and strain, unique accession number if available, and source.	Not Applicable	

Human research participants	Information included in the manuscript?	In which section is the information available? (Reagents and Tools Table, Materials and Methods, Figures, Data Availability Section)
If collected and within the bounds of privacy constraints report on age, sex and gender or ethnicity for all study participants.	Yes	Material and methods

Core facilities	Information included in the manuscript?	In which section is the information available? (Reagents and Tools Table, Materials and Methods, Figures, Data Availability Section)
If your work benefited from core facilities, was their service mentioned in the acknowledgments section?	Not Applicable	

Design

Study protocol	Information included in the manuscript?	In which section is the information available? (Reagents and Tools Table, Materials and Methods, Figures, Data Availability Section)
If study protocol has been **pre-registered, provide DOI in the manuscript**. For clinical trials, provide the trial registration number **OR** cite DOI.	Not Applicable	
Report the **clinical trial registration number** (at ClinicalTrials.gov or equivalent), where applicable.	Yes	Materials and Methods

Laboratory protocol	Information included in the manuscript?	In which section is the information available? (Reagents and Tools Table, Materials and Methods, Figures, Data Availability Section)
Provide DOI OR other citation details if **external detailed step-by-step protocols** are available.	Not Applicable	

Experimental study design and statistics	Information included in the manuscript?	In which section is the information available? (Reagents and Tools Table, Materials and Methods, Figures, Data Availability Section)
Include a statement about **sample size** estimate even if no statistical methods were used.	Yes	Figures
Were any steps taken to minimize the effects of subjective bias when allocating animals/samples to treatment (e.g. **randomization procedure**)? If yes, have they been described?	Yes	Figures, Materials and Methods
Include a statement about **blinding** even if no blinding was done.	Yes	Materials and Methods
Describe **inclusion/exclusion criteria** if samples or animals were excluded from the analysis. Were the criteria pre-established?<br><br>If sample or data points were omitted from analysis, report if this was due to attrition or intentional exclusion and provide justification.	Not Applicable	
For every figure, are **statistical tests** justified as appropriate? Do the data meet the assumptions of the tests (e.g., normal distribution)? Describe any methods used to assess it. Is there an estimate of variation within each group of data? Is the variance similar between the groups that are being statistically compared?	Yes	Figure legends

Sample definition and in-laboratory replication	Information included in the manuscript?	In which section is the information available? (Reagents and Tools Table, Materials and Methods, Figures, Data Availability Section)
In the figure legends: state number of times the experiment was **replicated** in laboratory.	Yes	Figures
In the figure legends: define whether data describe **technical or biological replicates**.	Yes	Figures

Ethics

Ethics	Information included in the manuscript?	In which section is the information available? (Reagents and Tools Table, Materials and Methods, Figures, Data Availability Section)
Studies involving **human participants**: State details of **authority granting ethics approval** (IRB or equivalent committee(s), provide reference number for approval.	Yes	Materials and Methods
Studies involving **human participants**: Include a statement confirming that **informed consent** was obtained from all subjects and that the experiments conformed to the principles set out in the WMA Declaration of Helsinki and the Department of Health and Human Services Belmont Report.	Yes	Materials and Methods
Studies involving **human participants:** For publication of **patient photos**, include a statement confirming that consent to publish was obtained.	Not Applicable	
Studies involving experimental **animals**: State details of **authority granting ethics approval** (IRB or equivalent committee(s), provide reference number for approval. Include a statement of compliance with ethical regulations.	Yes	Materials and Methods
Studies involving **specimen and field samples:** State if relevant **permits** obtained, provide details of authority approving study; if none were required, explain why.	Not Applicable	

Dual Use Research of Concern (DURC)	Information included in the manuscript?	In which section is the information available? (Reagents and Tools Table, Materials and Methods, Figures, Data Availability Section)
Could your study fall under dual use research restrictions? Please check biosecurity documents and list of **select agents and toxins** (CDC): https://www.selectagents.gov/sat/list.htm	Not Applicable	
If you used a select agent, is the security level of the lab appropriate and reported in the manuscript?	Not Applicable	
If a study is subject to dual use research of concern regulations, is the name of the **authority granting approval and reference number** for the regulatory approval provided in the manuscript?	Not Applicable	

Reporting

The MDAR framework recommends adoption of discipline-specific guidelines, established and endorsed through community initiatives. Journals have their own policy about requiring specific guidelines and recommendations to complement MDAR.

Adherence to community standards	Information included in the manuscript?	In which section is the information available? (Reagents and Tools Table, Materials and Methods, Figures, Data Availability Section)
State if relevant guidelines or checklists (e.g., **ICMJE, MIBBI, ARRIVE, PRISMA**) have been followed or provided.	Not Applicable	
For **tumor marker prognostic studies,** we recommend that you follow the **REMARK** reporting guidelines (see link list at top right). See author guidelines, under 'Reporting Guidelines'. Please confirm you have followed these guidelines.	Not Applicable	
For **phase II and III randomized controlled trials**, please refer to the **CONSORT** flow diagram (see link list at top right) and submit the CONSORT checklist (see link list at top right) with your submission. See author guidelines, under 'Reporting Guidelines'. Please confirm you have submitted this list.	Not Applicable	

Data Availability

Data availability	Information included in the manuscript?	In which section is the information available? (Reagents and Tools Table, Materials and Methods, Figures, Data Availability Section)
Have **primary datasets** been deposited according to the journal's guidelines (see 'Data Deposition' section) and the respective accession numbers provided in the Data Availability Section?	Not Applicable	
Were **human clinical and genomic datasets** deposited in a public access-controlled repository in accordance to ethical obligations to the patients and to the applicable consent agreement?	Not Applicable	
Are **computational models** that are central and integral to a study available without restrictions in a machine-readable form? Were the relevant accession numbers or links provided?	Not Applicable	
If publicly available data were reused, provide the respective **data citations in the reference list**.	Not Applicable	
